# Delimiting Andean catfish species of the families Astroblepidae and Loricariidae (Ostariophysi, Siluriformes) in the Peruvian Andes using DNA barcodes

**DOI:** 10.3897/zookeys.1290.182593

**Published:** 2026-08-18

**Authors:** Aaron De la Sota, Max Hidalgo, Guillain Estivals, Dario Faustino-Fuster, Vanessa Meza-Vargas, Pierre Caminade, Leah Bêche, Ricardo Britzke R, Dirk Steinke, Nicolas Hubert

**Affiliations:** 1 Departamento de Ictiología, Museo de Historia Natural, Universidad Nacional Mayor de San Marcos, Lima, Peru Departamento de Ictiología, Museo de Historia Natural, Universidad Nacional Mayor de San Marcos Lima Peru; 2 ISEM, Université de Montpellier, CNRS, IRD, Montpellier, France Laboratorio de Biología y Genética Molecular (LBGM), Instituto de Investigaciones de la Amazonía Peruana (IIAP) Iquitos Peru; 3 Laboratorio de Biología y Genética Molecular (LBGM), Instituto de Investigaciones de la Amazonía Peruana (IIAP), Iquitos, Peru Observatorio de la Biodiversidad de la Amazonia Peruana (OBAP), Instituto de Investigaciones de la Amazonia Peruana (IIAP) Iquitos Peru; 4 Observatorio de la Biodiversidad de la Amazonia Peruana (OBAP), Instituto de Investigaciones de la Amazonia Peruana (IIAP), Iquitos, Peru Centre for Biodiversity Genomics, University of Guelph Guelph Canada; 5 Observatorio de la Biodiversidad de la Amazonia Peruana (OBAP), Institut de Recherche pour le Développement (IRD), Marseille, France Centre d'Ingénierie Hydraulique (CIH) La Motte Servolex France; 6 Centre d'Ingénierie Hydraulique (CIH), Electricité de France, 4 Allées du Lac des Tignes, 73290 La Motte Servolex, France ISEM, Université de Montpellier Montpellier France; 7 Centre for Biodiversity Genomics, University of Guelph, 50 Stone Rd E, Guelph, ON N1G2W1, Canada Observatorio de la Biodiversidad de la Amazonia Peruana (OBAP), Institut de Recherche pour le Développement (IRD) Marseille France

**Keywords:** Altitudinal gradient, Amazon, Andes, cryptic diversity, DNA barcoding, species turn-over

## Abstract

The Amazon Basin, the largest on Earth, hosts an exceptionally rich fish fauna shaped by its ancient evolutionary history, vast environmental heterogeneity, and steep altitudinal gradient. Despite this diversity, major taxonomic gaps persist, including the Linnean shortfall—the large number of undescribed species—and the Wallacean shortfall, reflecting limited knowledge of species distributions. During the past two decades, DNA barcoding using the mitochondrial COI gene has helped uncover substantial cryptic diversity in Amazonian fishes, particularly along the Andean slope, where Siluriformes exhibit striking levels of undocumented diversity. In this study, we investigated catfish communities from the families Astroblepidae and Loricariidae in the headwaters of the Madre de Dios and Huallaga rivers in Peru, spanning elevations from 500 to 2,500 m. Using DNA barcoding and multiple species-delimitation methods, we identified 27 MOTUs, most of which could not be assigned to recognized species. Our aim was to examine diversity patterns, species co-occurrence, and phylogenetic community structure along the Andean slope. This framework allows us to evaluate how ecological processes—such as competition, niche partitioning, and ecological specialization—shape community assembly. Combined with evidence of close phylogenetic relationships among MOTUs, our results highlight the likely importance of *in situ* diversification in driving species turnover and structuring catfish biodiversity across Andean elevational gradients.

## Introduction

The Amazon is the largest river on Earth, covering more than 6 million km^2^ (Venticinque et al. 2016). It hosts the richest ichthyofauna in the world, with more than 3,500 fish species spending at least part of their life cycle within its watershed (Reis et al. 2003; Jézéquel et al. 2020). This remarkable diversity is attributed to several factors, including the ancient origin of the Neotropical fish fauna (Lenglin et al. 2025), a complex biogeographic history (Hubert and Renno 2006; Albert and Reis 2011) and a wide variety of environmental conditions (Sioli 2012; Junk 2013). Another factor accounting for its exceptional diversity is the remarkable length of the river (nearly 7,000 km) and the extreme altitudinal gradient associated with it, ranging from 5,000 meters to the sea, and responsible for idiosyncratic fish diversity patterns (Fernandes et al. 2004; Hubert and Renno 2006; Oberdorff et al. 2019). This complex framework likely accounts for the major knowledge gaps in the diversity of Amazonian fishes, which arguably hinder science-based conservation efforts in the region as well as the development of identification tools for monitoring these species. This situation is currently referred to as the taxonomic impediment, which describes the lack of taxonomic knowledge and expertise that limits the advancement of biodiversity research (Giangrande 2003). The taxonomic impediment involves two major knowledge gaps. First is that a substantial portion of Amazonian fish species remain undescribed, a phenomenon known as the Linnean shortfall (Brito 2010; Wani et al. 2024). Second is that the geographic distributions of most species are poorly known, a gap referred to as the Wallacean shortfall (Bini et al. 2006; Wani et al. 2024).

During the past two decades, major advancements in molecular methods have enabled researchers to circumvent both the Linnean and Wallacean shortfalls. One key development is DNA barcoding, the use of the mitochondrial gene of the Cytochrome c oxidase I (COI) as an internal species tag for animals (Hebert et al. 2003). Designed as a universal system for assigning unknown specimens to known species, the first step involves developing reference libraries from adult specimens identified using diagnostic morphological characters (Hajibabaei et al. 2007; Hubert et al. 2008). Initial studies in tropical faunas quickly revealed that both the Linnean and Wallacean shortfalls were major obstacles to the development of these reference libraries (Hebert et al. 2004; Smith et al. 2008). Since then, DNA barcoding, together with species-delimitation methods, has been included in taxonomic workflows as an efficient approach for the initial screening of biodiversity during inventories of poorly known faunas (Janzen et al. 2005; Kekkonen and Hebert 2014; Delrieu-Trottin et al. 2025). The use of DNA barcoding for inventories has been successfully applied to freshwater fish fauna in various regions (Hubert et al. 2008; Pereira et al. 2013; Knebelsberger et al. 2015; Dahruddin et al. 2017; Sonet et al. 2019; Sholihah et al. 2020). Initial studies applying DNA barcoding to Amazonian fishes revealed substantial levels of both undescribed or cryptic diversity, underscoring the extent to which actual species richness has been underestimated in many taxonomic groups (Pereira et al. 2011, 2013; Castro Paz et al. 2014; Benzaquem et al. 2015; Rossini et al. 2016; Machado et al. 2018). However, the Linnean shortfall is even more pronounced in the lineages distributed along the Andean slope, where the use of mitochondrial DNA sequencing has revealed substantial proportions of unknown diversity in multiple groups, particularly among catfish families such as Astroblepidae and Loricariidae (Schaefer et al. 2011; de Borba et al. 2019; Ochoa et al. 2020; Meza-Vargas et al. 2024). Currently, eco-evolutionary dynamics underlying diversification and spatial species turn-over along the Andean slope are still poorly understood (Lujan et al. 2013).

**Figure 1.**
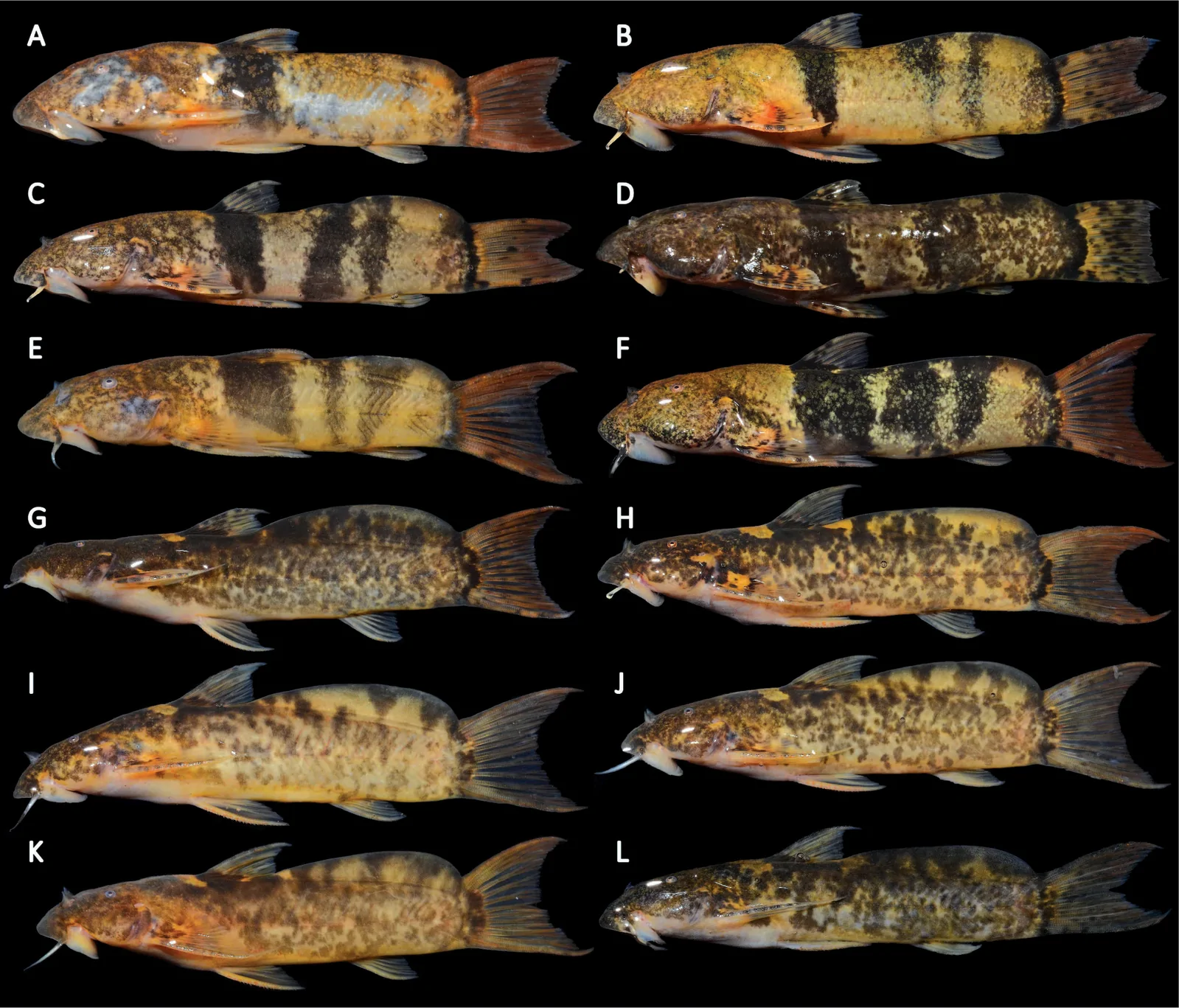
Phenotypic variability and variations in coloration pattern of A–F. Astroblepus huallagaensis; G–L. Astroblepus sp. (BOLD:AER0687).

**Figure 2.**
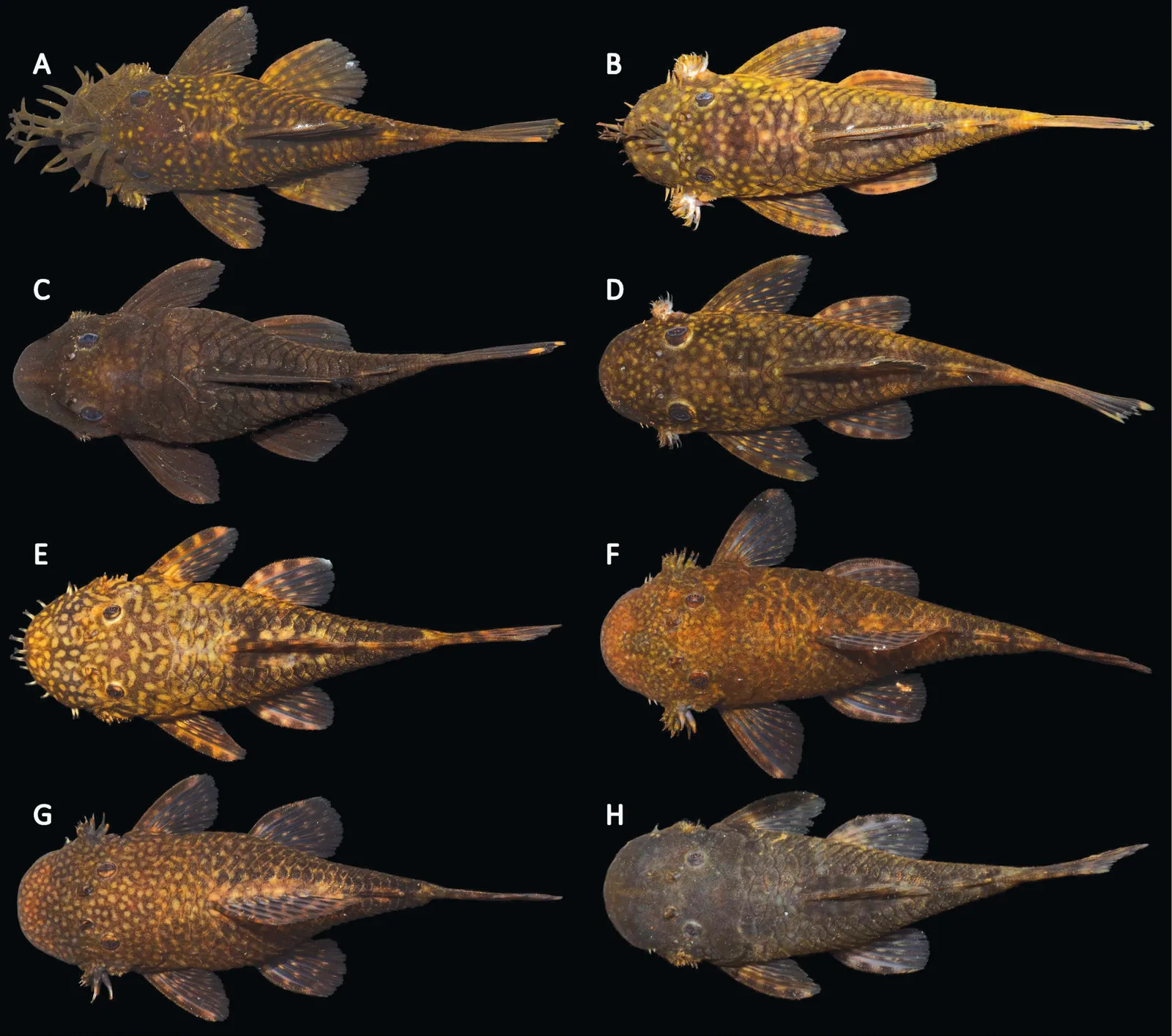
Phenotypic variability and variations in coloration pattern of *Ancistrus* sp. (BOLD:AER0500; **A–D**) and *Ancistrus maldonadoi* (**E–H**).

**Figure 3.**
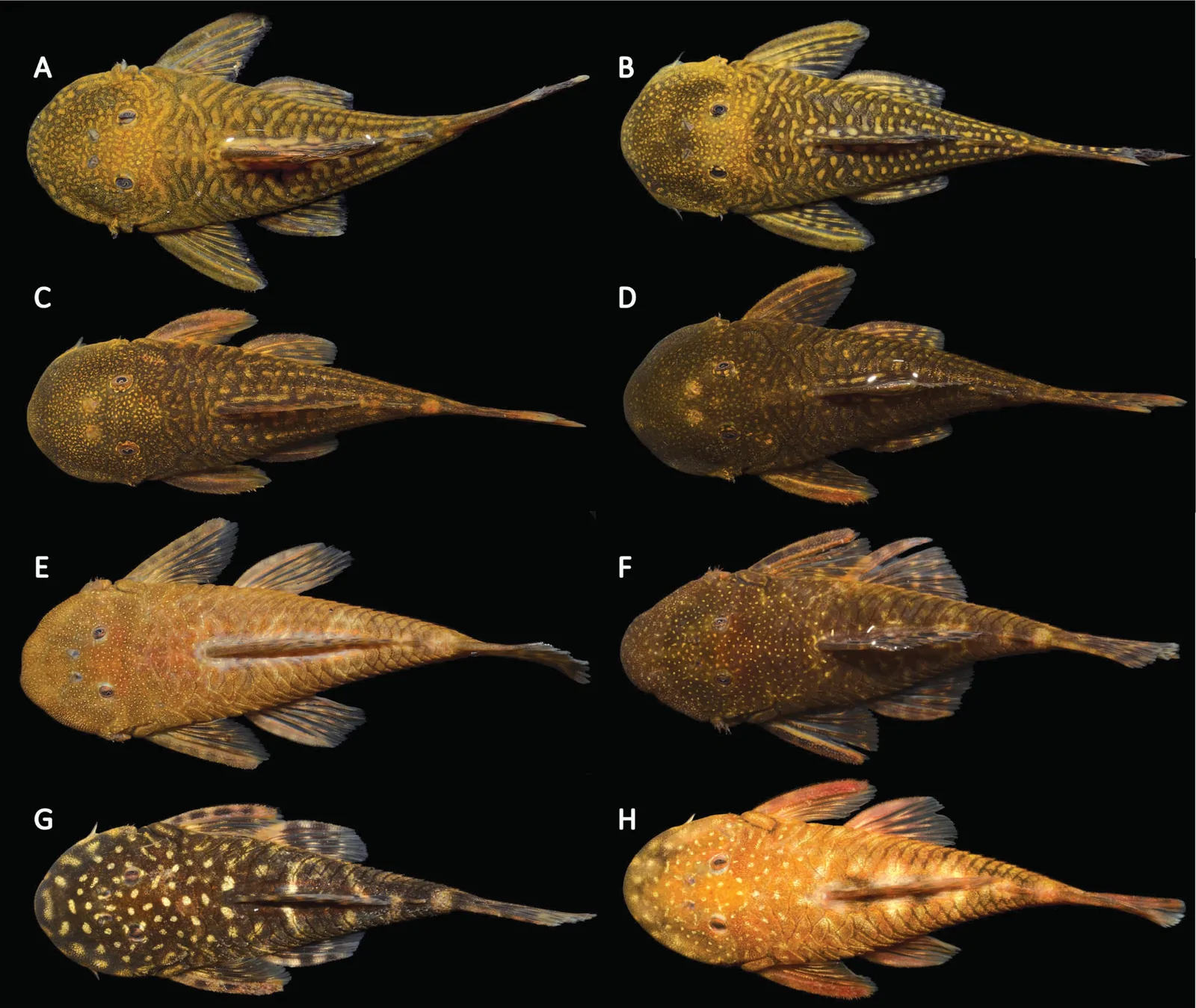
Phenotypic variability and variations in coloration pattern of *Chaetostoma branickii* (**A–D**) and *Chaetostoma microps* (**E–H**).

The families Astroblepidae and Loricariidae are among the most species-rich and ecologically dominant catfish groups along the Andean slope (Jacobsen 2008; Lujan et al. 2013; Carvajal‐Quintero et al. 2015; Roxo et al. 2019; Miranda et al. 2022). The family Astroblepidae includes a single genus, *Astroblepus*, with 82 valid species restricted to the Andes (Fricke et al. 2026). The group is distributed from Panama to Bolivia, with highest diversity in Colombia, Ecuador, and Peru (Froese and Pauly 2026). *Astroblepus* species are generally small- to medium-sized catfishes, typically not exceeding 25 cm standard length, and inhabit fast-flowing, high-elevation streams between 500 and more than 4,000 meters. They are highly specialized, possessing adhesive mouthparts that allow them to cling to substrates and even climb waterfalls in steep Andean environments (Jacobsen 2008). Approximately 20–22 of the 82 valid species of *Astroblepus* are currently reported from Peru (Fricke et al. 2026), and several additional candidate taxa have been identified in the literature based on mitochondrial sequences data (Schaefer et al. 2011; Ochoa et al. 2020). Uncertainties in the taxonomy of the genus likely stem from the paucity of diagnostic external characters, as well as pronounced intraspecific variation in coloration across sites and size classes (Fig. 1). Loricariidae is the most diverse groups of freshwater catfishes in South America with 1,083 valid species (Fricke et al. 2026). They are characterized by bony dermal plates covering the body and a ventral suckermouth adapted for attachment to rocks in fast-flowing rivers. Recent molecular phylogenetic studies have significantly revised the classification of the family, revealing that several traditional genera were not monophyletic, leading to taxonomic revisions and the description of new Andean genera (Montoya-Burgos et al. 1998; Armbruster 2004; Chiachio et al. 2008; Lujan et al. 2015). In the Andes, the family is mostly represented by the genera *Ancistrus* and *Chaetostoma*. *Ancistrus* species, often called bristlenose plecos, are recognized by the fleshy tentacles present on the snout of adult males (Fig. 2). The genus includes ~ 80 species (Froese and Pauly 2026), which inhabit a wide variety of freshwater habitats across South America and exhibit considerable ecological and morphological diversity. *Chaetostoma*, known as rubbernose plecos, is particularly associated with the Andes. This genus includes ~ 50 species distributed from Panama to southern Peru along both Pacific and Atlantic Andean slopes (Froese and Pauly 2026). *Chaetostoma* species are adapted to cold, oxygen-rich mountain streams with strong currents. Their enlarged oral disks and flattened bodies allow them to cling to rocks while grazing on algae and detritus (Fig. 3). The Andean region has played a major role in loricariid diversification because mountain uplift created isolated river systems that promoted speciation and endemism (Montoya-Burgos et al. 1998; Armbruster 2004; Chiachio et al. 2008; Lujan et al. 2015). In this context, the two families constitute well-suited models for exploring the dynamics of species turnover and diversification underlying the assembly of fish communities and patterns of endemism in the Andes.

In the present study, we sampled catfish species from the family Astroblepidae and Loricariidae in headwaters of the Madre de Dios and Huallaga rivers in Peru, from an altitude ranging 500–2500 m, to examine patterns of diversity and spatial species turn-over along the Andean slope, and investigate potential mechanisms of spatial species segregation related to ecological specialization and niche partitioning. Because of the remarkable phenotypic diversity in *Astroblepus*, *Ancistrus* and *Chaetostoma* species (Figs 1, 2, 3), the community composition of these catfish assemblages was examined across 34 sites through DNA barcoding and species delimitation methods with the following objectives: explore diversity and species co-occurrence patterns to detect potential departures from random species aggregation that may result from biotic interactions such as competitive interactions (Hutchinson 1959; Macarthur and Levins 1967) or life history trade-offs (MacArthur and Wilson 1967), and explore phylogenetic community structure in order to detect a potential departure of the distribution of cladogenetic events from expected at random that may result from the overlap of speciation dynamics and species aggregation in ecological communities (Gillespie 2004; Emerson and Gillespie 2008; Hubert et al. 2015).

## Material and methods

### Sampling and voucher specimens

**Figure 4.**
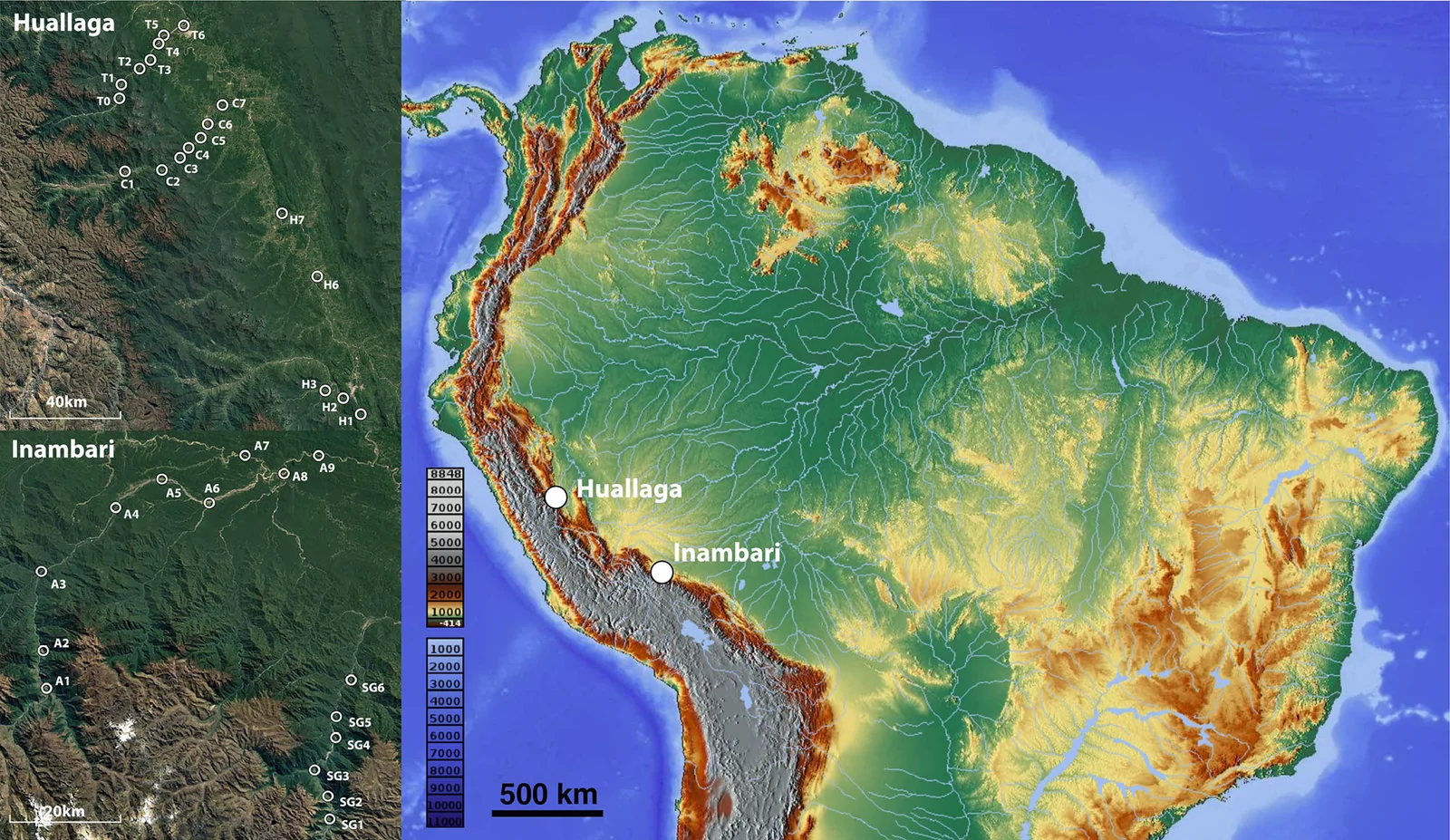
Map of the study area displaying the 15 sampling sites in the upper Inambari and the 19 sampling sites in the Upper Huallaga.

Specimens were captured using electrofishing, seine nets, and cast nets across 34 sites in Peru (Fig. 4). Specimens were identified following original descriptions, and valid names were further checked using online catalogs (Fricke et al. 2026; Froese and Pauly 2026). Specimens were photographed, individually labeled, and voucher specimens were preserved in a 5% formalin solution before transferring to a 70% ethanol solution. A fin clip or a muscle biopsy was taken for each specimen and fixed in a 95% ethanol solution for further genetic analyses. Tissues and voucher specimens were deposited at the Department of Ichthyology from the Museo de Historia Natural - Universidad Nacional Mayor de San Marcos (**MUSM**).

*Astroblepus* species were identified based on the relative length of the maxillary barbels compared with the suction disc, the length of the first pectoral-fin ray, the number of soft rays in the pectoral fin, the length of the first anal-fin ray relative to the anus, the number of rows of bicuspid teeth on the premaxilla, the position of the pectoral-fin spine, and the shape of the adipose fin (Regan 1904; Ardila Rodríguez 2012, 2015).

The genera *Ancistrus* and *Chaetostoma* were distinguished based on the shape of the snout and the presence of an inflated margin, the presence of fleshy tentacles on the snout, the presence of odontodes in the cheek, the size of the maxillary barbels, the shape of the opercular eye opening and its position relative to the anterior margin of the opercle, the number of plate rows on the caudal peduncle, the number of predorsal plate rows, the number of branched rays in the dorsal fin, and jaw shape and tooth number (Regan 1904; Armbruster 2004; Maldonado-Ocampo et al. 2005; Salcedo 2006; van der Sleen and Albert 2017; Bifi et al. 2019).

*Chaetostoma* species were distinguished based on the presence of light spots or dark lines on the body, varying in shape and coloration, overall body shape, the number of cheeks odontodes, the number of branched rays in the dorsal fin, the presence of a supraoccipital crest, the presence of rounded papillae on the snout, and the relative position of the dorsal fin in relation to the anterior margin of the preadipose plate (Eigenmann 1942; Salcedo 2006; Lujan et al. 2015; Meza-Vargas et al. 2024).

*Ancistrus* species were distinguished based on the shape, coloration, and number of light spots on the body, the number of cheek spines, the shape of the snout and the width of its margin, the number and shape of the snout tentacles, the presence of a keel formed by preadipose plates, orbital diameter size, tooth shape, and dentary width (Kettleborough et al. 1924; Fowler 1945; Isbrücker et al. 2001; Taphorn et al. 2013; Bifi and Ortega 2020).

### DNA extraction, amplification, and sequencing

Total genomic DNA was extracted using the Qiagen DNeasy 96 tissue extraction kit following the manufacturer’s specifications. For all specimens, a 652-bp segment from the 5’ region of the cytochrome c oxidase I gene (COI) was amplified using C_FishF1t1/C_FishR1t1 primers including M13 tails (Ivanova et al. 2007). PCR amplifications were performed using a Veriti 96-well Fast (ABI-AppliedBiosystems) thermocycler in a final volume of 10.0 μl containing 5.0 μl Buffer 2X, 3.3 μl ultrapure water, 1.0 μl each primer (10 μM), 0.2 μl enzyme Phire® Hot Start II DNA polymerase (5U) and 0.5 μl of DNA template (~50 ng). Amplifications employed the following thermocycling regime: initial denaturation at 98 °C for 5 min followed by 30 cycles denaturation at 98 °C for 5 s, annealing at 56 °C for 20 s and extension at 72 °C for 30 s, followed by a final extension step at 72 °C for 5 min. The PCR products were purified with ExoSap-IT® (USB Corporation, Cleveland, OH, USA) and sequenced in both directions. Sequencing reactions were performed using the BigDye® Terminator v. 3.1 Cycle Sequencing Ready Reaction” and sequencing was performed on an automatic sequencer ABI 3130 DNA Analyzer (Applied Biosystems). Sequences and collateral information (photographs, voucher collection number, and collection data) were deposited in BOLD (Ratnasingham and Hebert 2007) at dx.doi.org/10.5883/DS-ANDEAN and GenBank.

### Genetic species delimitation and phylogenetic reconstructions

The inventory of Molecular Operational Taxonomic Units (MOTUs), defined as diagnosable mitochondrial lineages, was conducted using five methods of species delimitation including: (1) Refined Single Linkage (RESL), implemented in BOLD to produce Barcode Index Numbers (BINs) (Ratnasingham and Hebert 2013), (2) Poisson Tree Process (PTP) (Zhang et al. 2013) in its single (sPTP) and (3) multiple rate version (mPTP) available at https://mptp.h-its.org/, and (4) General Mixed Yule-Coalescent (GMYC) in its single (sGMYC) and (5) multiple rate version (mGMYC) as implemented in the R package Splits 1.0-19 (Fujisawa and Barraclough 2013). The final delimitation scheme, used to delineate MOTUs, was established following a majority-rule consensus among the six methods (Shen et al. 2019; Sholihah et al. 2020; Arida et al. 2021).

The RESL algorithm use DNA alignments as input. For PTP analyses, a Maximum Likelihood (ML) tree was generated using IQ‐TREE (Nguyen et al. 2015) with the most-likely substitution model according to ModelFinder following the Bayesian Information Criterion (BIC) (Kalyaanamoorthy et al. 2017) available at http://iqtree.cibiv.univie.ac.at (Trifinopoulos et al. 2016). Finally, the ultrametric and fully resolved tree required by GMYC analyses was reconstructed using the Bayesian approach implemented in BEAST 2.4.8 (Bouckaert et al. 2014). Two Markov chains of 50 million each were run independently using a Yule pure birth model tree prior, a strict-clock model of 1.2% of genetic distance per million years (Bermingham et al. 1997), and a GTR + I + Γ substitution model. Trees were sampled every 10,000 states after an initial burn-in period of 10 million. Both runs were combined using LogCombiner 2.4.8, and the maximum credibility tree was established using TreeAnnotator 2.4.7 (Bouckaert et al. 2014). Sequences were collapsed into haplotypes prior to Bayesian analyses.

Once MOTUs were delimited, DNA barcodes were used concomitantly to reconstruct phylogenetic trees using the StarBEAST2 package (Ogilvie et al. 2017) in BEAST 2.4.8. StarBEAST2 jointly reconstructs gene trees and MOTU trees using Bayesian inference under a combined Yule-coalescent framework (Fujiwasa and Barraclough 2013; Ogilvie et al. 2017). This approach infers branching times and topologies among MOTU under a Yule speciation model, and among haplotypes within MOTUs under a coalescent model (Kingman 1982). Consequently, this Yule-coalescent model requires prior designation of MOTUs, which were defined here using a majority rule consensus of the lineage delimitation analyses. Analyses were conducted using a single partition including protein-coding regions, a GTR+I+T substitution model, an Uncorrelated Log Normal (UCLN) species tree model, and MCMC of 20 million steps long. Divergence time estimates were inferred across the tree using a relaxed lognormal molecular clock model and a substitution rate of 1.2% per million years, as mentioned above. Independent runs were combined using LogCombiner 1.10. Gene and MOTUs maximum clade credibility trees, age estimates and 95% highest posterior density (HPD) intervals were summarized using TreeAnnotator 1.10.4.

### Species turn-over and phylogenetic community structure

MOTU occurrences were recorded across the 34 sampling sites (Fig. 4). A hierarchical clustering of sites was established based on dissimilarities in species occurrence across sites, using the R package *vegan* (Oksanen et al. 2007), and the average phylogenetic distance among species across sites using the R package picante (Kembel et al. 2010). Patterns of species composition within sites were examined using the Simpson dominance index *D* (Simpson 1949), the Margalef index *d* (Margalef 1973) and the Pielou index *J* (Pielou 1966) as implemented in Past 3.0 (Hammer and Harper 2001). Patterns of species aggregation were examined using the probabilistic model developed by Veech (Veech 2013), as implemented in the *cooccur* package in R (Griffith et al. 2016). This approach estimates the expected probabilities of species co-occurrence under a null model of random associations, allowing the detection of significant departures indicative of species repulsion or positive association dynamics. The signature of non-random dynamics of species co-occurrence on phylogenetic relatedness within communities was further investigated by calculating the Mean Phylogenetic Distance (i.e., MPD, the average phylogenetic distance among species within a site) and the Mean Nearest Taxon Distance (i.e., MNTD, the average phylogenetic distance between each species and its closest relative within a site), as implemented in the *picante* package in R (Kembel et al. 2010). Departures of phylogenetic distances from those expected under random patterns of species co-occurrence were assessed using 1,000 random samplings of species from the regional pool.

## Results

**Figure 5.**
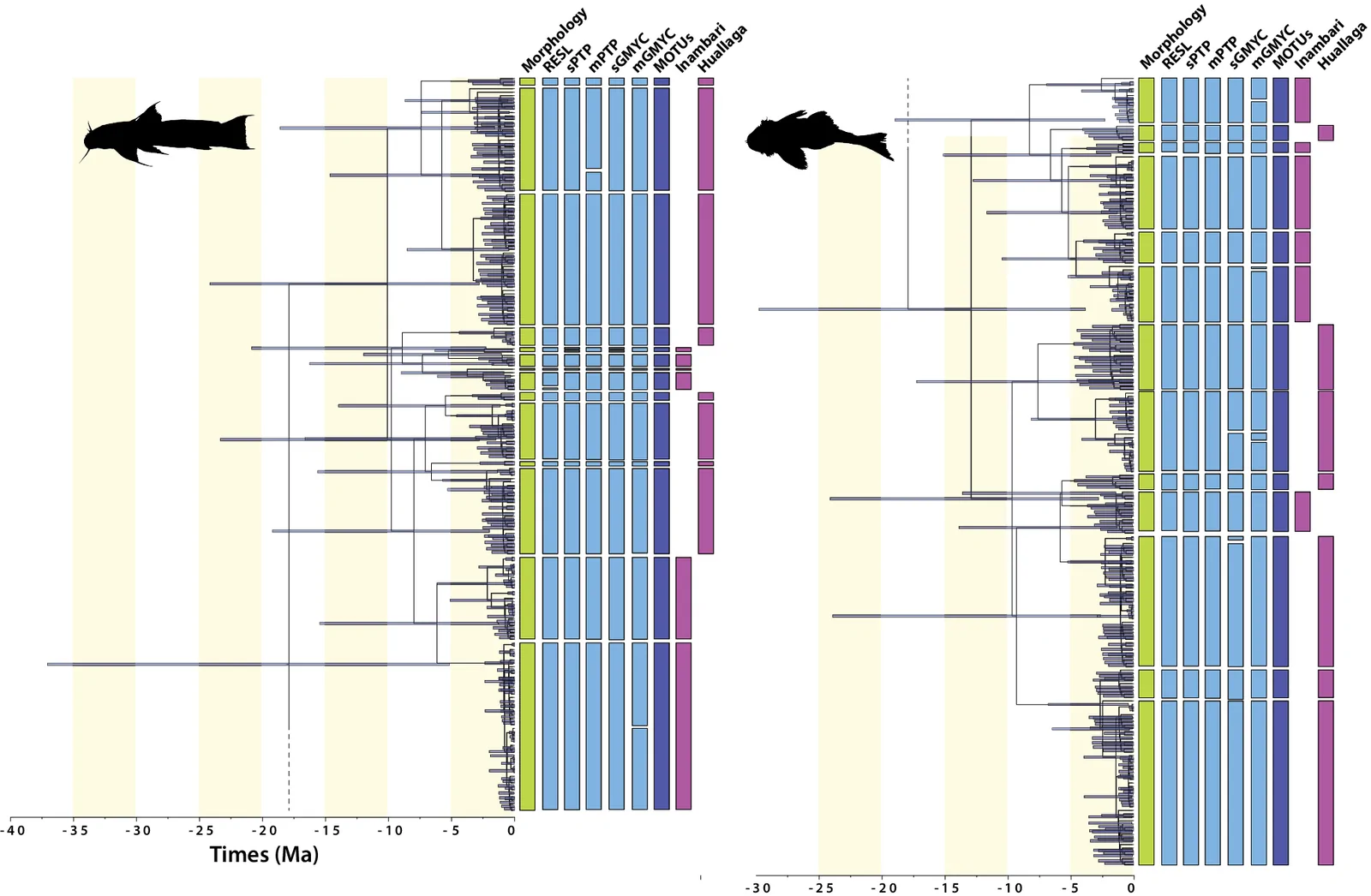
Bayesian mitochondrial gene tree based on 446 COI sequences reconstructed using the SpeciesTree UCLN algorithm in BEAST 2.4.8. The 95% Highest Posterior Density (HPD) intervals for node age estimates are shown as horizontal bars. The figure also presents the results of five MOTU delimitation methods—sGMYC, mGMYC, sPTP, mPTP, and RESL (light blue)—along with species delimitation based on morphological characters (light green) and the majority-rule consensus (dark blue).

**Figure 6.**
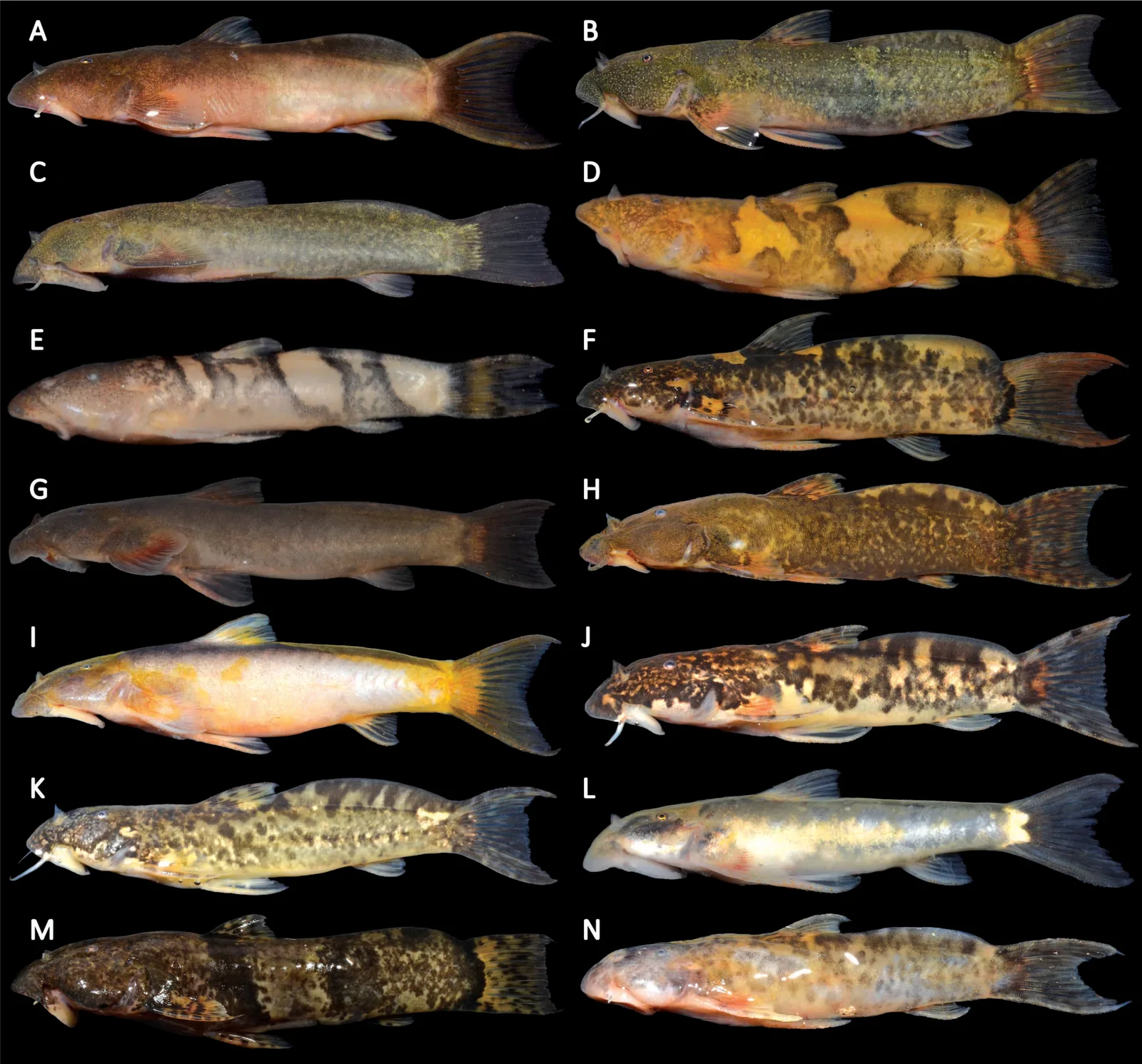
Photographs of the 14 MOTUs delimited in the genus Astroblepus including **A**. *Astroblepus* sp. (BOLD:AAM2932); **B**. *Astroblepus* sp. (BOLD:AAM2933); **C**. *Astroblepus* sp. (BOLD:AAN2234); **D**. *Astroblepus* sp. (BOLD:AER0685); **E**. *Astroblepus* sp. (BOLD:AER0686); **F**. *Astroblepus* sp. (BOLD:AER0687, MOTU07); **G**. *Astroblepus* sp. (BOLD:AER0687, MOTU17); **H**. *Astroblepus* sp. (BOLD:AGY9904); **I**. *Astroblepus* sp. (BOLD:AGY9906); **J**. *Astroblepus* sp. (BOLD:AGY9907); **K**. *Astroblepus* sp. (BOLD:AGY9908); **L**. *Astroblepus quispei*; **M**. *Astroblepus huallagaensis*; **N**. *Astroblepus* sp. (BOLD:AGY9903).

**Figure 7.**
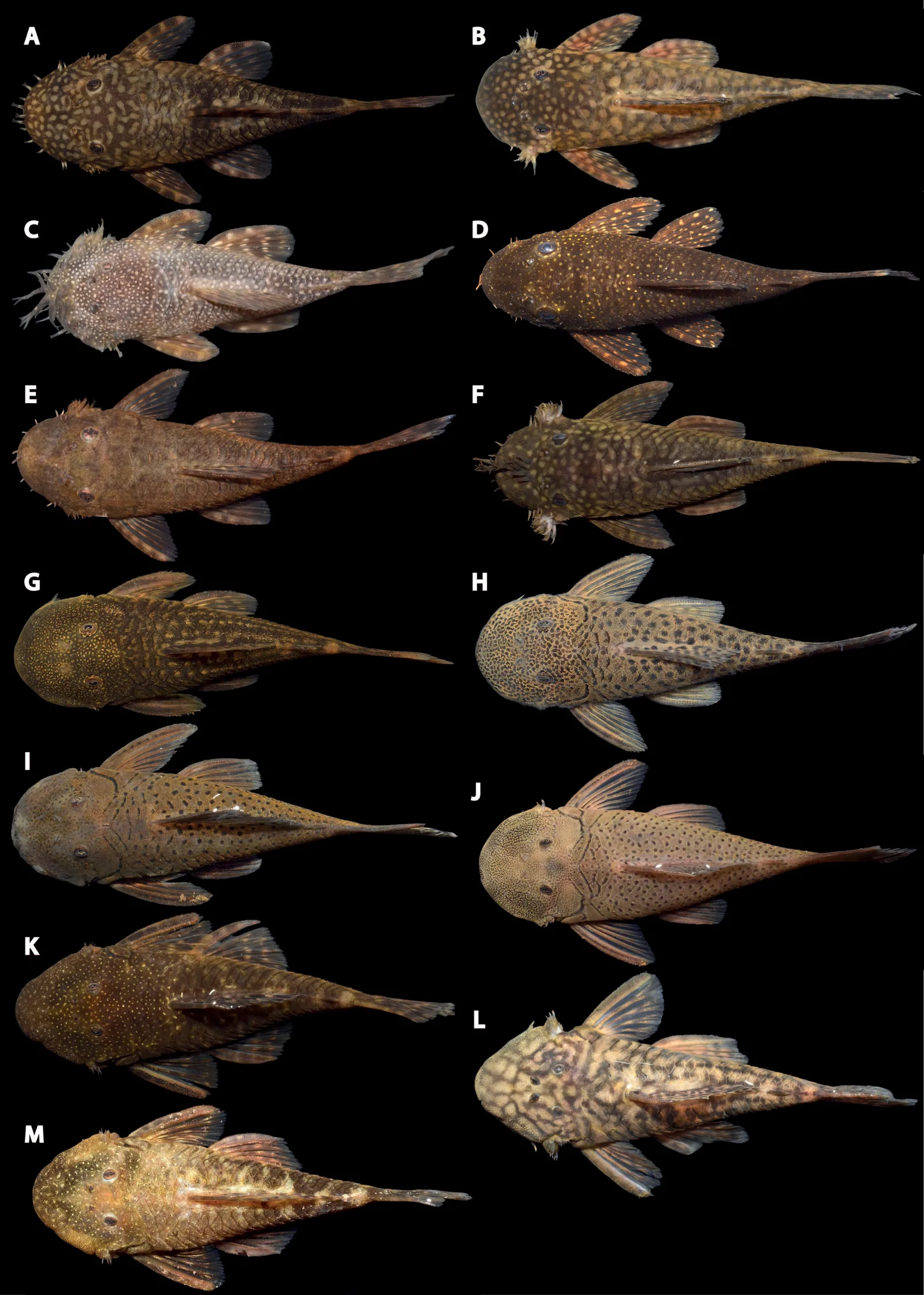
Photographs of the 13 MOTUs delimited in the genera *Ancistrus* (**A–F**) and *Chaetostoma* (**G–M**) including **A**. *Ancistrus maldonadoi*; **B**. *Ancistrus marcapatae*; **C**. *Ancistrus megalostomus*; **D**. *Ancistrus tamboensis*; **E**. *Ancistrus* sp. (BOLD:AER0499); **F**. *Ancistrus* sp. (BOLD:AER0500); **G**. *Chaetostoma branickii*; **H**. *Chaetostoma daidalmatos*; **I**. *Chaetostoma lineopunctatum* (MOTU06); **J**. *Chaetostoma lineopunctatum* (MOTU16); **K**. *Chaetostoma microps*; **L**. *Chaetostoma stroumpoulos*; **M**. *Chaetostoma* sp. (BOLD:ACM9082).

A total of 446 DNA barcodes were successfully generated, including 215 and 231 sequences for the families Loricariidae and Astroblepidae, respectively, and 168 and 278 sequences from the Araza and Huallaga rivers, respectively (Suppl. material 2). All the sequences were longer than 500 bp, and no stop codons were detected, indicating that the collected sequences represent functional coding regions. DNA-based species delimitation methods produced congruent results, yielding 24 MOTUs with mPTP, 29 MOTUs with sPTP, 27 MOTUs with RESL, 28 MOTUs with sGMYC, and 30 MOTUs with mGMYC (Fig. 5; Suppl. material 2). The final consensus scheme consisted of 27 MOTUs (Fig. 5, Table 1), of which only 12 were identified to the species level based on morphological characters (Figs 6, 7). All MOTUs are distributed within a single watershed and tend to form phylogenetically clustered groups within each watershed. Maximum intraspecific distances within MOTUs identified to the species level ranged from 0 (*Ancistrus tamboensis*, *Chaetostoma lineopunctatum*, *Astroblepus huallagaensis* and *A. quispei*) to 2.56% (*Ancistrus megalostomus*) (Table 1). Minimum interspecific distances ranged from 1.14 (*Chaetostoma stroumpoulos*) to 7.53% (*Chaetostoma microps*) (Table 1). A total of 15 MOTUs were identified only to the genus level based on morphological characters, most of them belonging to the genus *Astroblepus*, which contained 12 unidentified MOTUs (Table 2). The genetic K2P distances observed within MOTUs or between the closest MOTUs were similar to those observed for MOTUs identified to the species level. Maximum intra-MOTUs K2P genetic distances ranged from 0–2.09%, and the distance to the nearest-neighbor ranged from 2.08–7.16%. The Bayesian phylogenetic reconstruction indicates that most MOTUs originated before 5 Ma in both families (Fig. 5).

**Table 1.** List of the nominal species identified using their morphological characters along with their MOTUs and BIN numbers, maximum K2P genetic distance within each MOTU (Distance max in %) and K2P genetic distance to the nearest neighbor (Distance NN, %).

**Family**	**Species**	**MOTU**	**BIN**	**Distance max (%)**	**Distance NN (%)**
Loricariidae	*Ancistrus tamboensis* (Fowler, 1945)	MOTU1	BOLD:ADG1133	0%	1.44%
Loricariidae	*Ancistrus maldonadoi* (Bifi & Ortega, 2020)	MOTU22	BOLD:AER0497	1.44%	2.72%
Loricariidae	*Ancistrus megalostomus* (Pearson, 1924)	MOTU23	BOLD:AER0498	2.56%	4.31%
Loricariidae	*Ancistrus marcapatae* (Regan, 1904)	MOTU26	BOLD:AER0501	0.96%	4.53%
Loricariidae	*Chaetostoma microps* (Gunter, 1864)	MOTU2	BOLD:ACM9082	1.61%	7.53%
Loricariidae	*Chaetostoma branickii* (Regan,1904)	MOTU3	BOLD:AGY8549	0%	3.55%
Loricariidae	*Chaetostoma stroumpoulos* (Salcedo, 2006)	MOTU4	BOLD:AGZ1150	0.65%	1.14%
Loricariidae	*Chaetostoma daidalmatos* (Salcedo, 2006)	MOTU5	BOLD:AGY2166	0.48%	2.88%
Loricariidae	*Chaetostoma lineopunctatum* (Eigenmann, 1942)	MOTU6	BOLD:AGY8550	0%	2.88%
Loricariidae	*Chaetostoma lineopunctatum* (Eigenmann, 1942)	MOTU16	BOLD:AFG1283	0.32%	2.08%
Astroblepidae	*Astroblepus huallagaensis* (Rodríguez, 2013)	MOTU8	BOLD:AGY2510	0%	2.08%
Astroblepidae	*Astroblepus quispei* (Ardila, 2012)	MOTU9	BOLD:AGY9905	0%	4.06%

**Table 2.** List of the unidentified MOTUs, including their family and genus assignments, consensus MOTU and BIN numbers, maximum K2P genetic distance within each MOTU (Distance max. in %), and K2P genetic distance to the nearest neighbor (Distance NN in %).

**Family**	**Genus**	**MOTU**	**BIN**	**Distance max (%)**	**Distance NN (%)**
Astroblepidae	* Astroblepus *	MOTU20	BOLD:AAM2933	2.09%	2.56%
Astroblepidae	* Astroblepus *	MOTU27	BOLD:AER0685	1.92%	5.92%
Astroblepidae	* Astroblepus *	MOTU13	BOLD:AGY9904	0.58%	4.67%
Astroblepidae	* Astroblepus *	MOTU19	BOLD:AAM2932	1.12%	4.48%
Astroblepidae	* Astroblepus *	MOTU21	BOLD:AAN2234	1.28%	2.88%
Astroblepidae	* Astroblepus *	MOTU18	BOLD:AER0686	0.16%	5.92%
Astroblepidae	* Astroblepus *	MOTU7	BOLD:AER0687	0.66%	2.08%
Astroblepidae	* Astroblepus *	MOTU17	BOLD:AER0687	0%	2.88%
Astroblepidae	* Astroblepus *	MOTU10	BOLD:AGY9903	0%	2.72%
Astroblepidae	* Astroblepus *	MOTU14	BOLD:AGY9906	1.47%	7.16%
Astroblepidae	* Astroblepus *	MOTU12	BOLD:AGY9907	0%	5.13%
Astroblepidae	* Astroblepus *	MOTU11	BOLD:AGY9908	0.32%	2.72%
Loricariidae	* Ancistrus *	MOTU25	BOLD:AER0500	0.32%	3.20%
Loricariidae	* Ancistrus *	MOTU24	BOLD:AER0499	0%	3.79%
Loricariidae	* Chaetostoma *	MOTU15	BOLD:ACM9082	0.34%	5.99%

**Figure 8.**
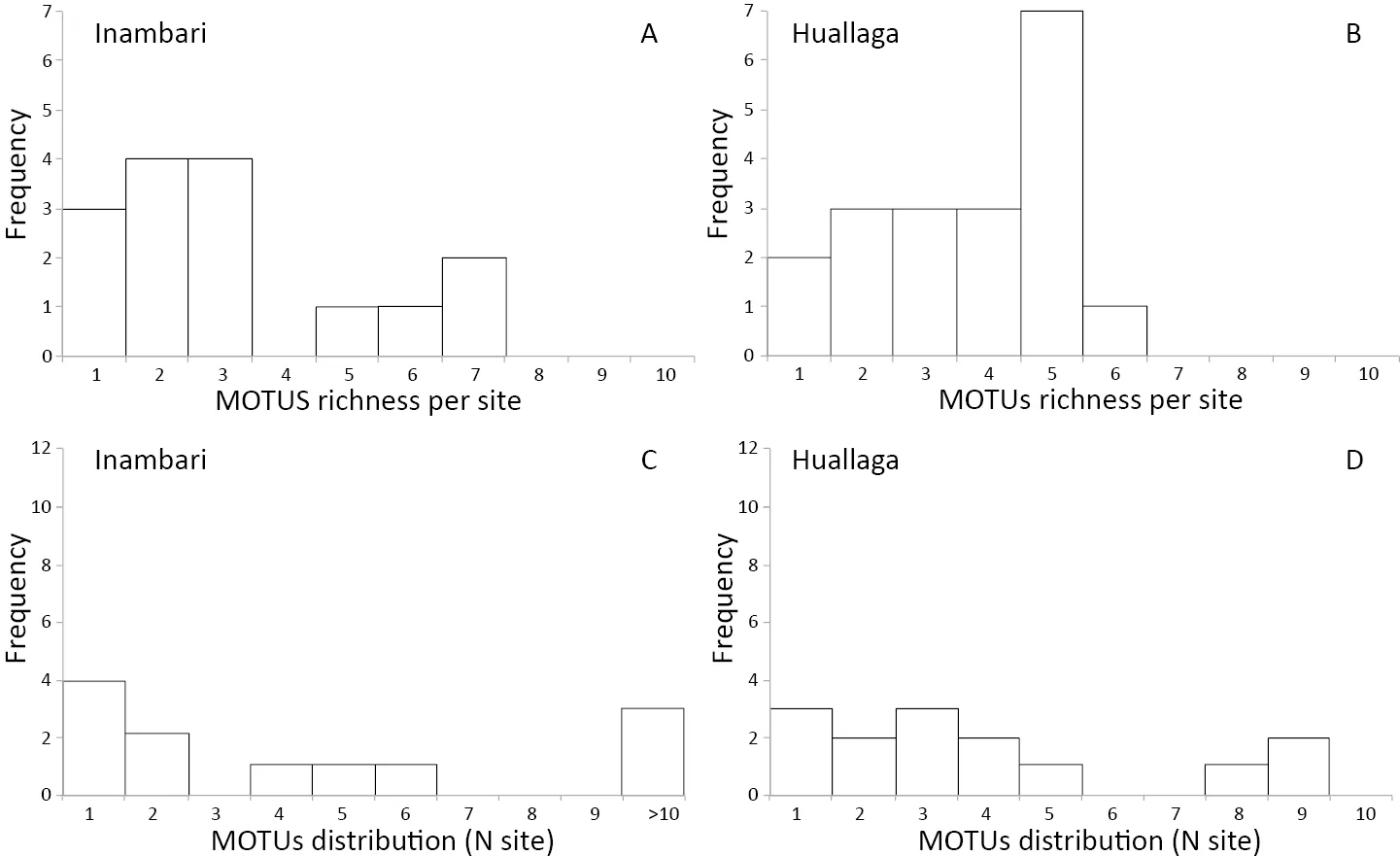
Distribution of MOTUs richness per site within the Inambari and Huallaga, **A**, **B** respectively, and distribution of MOTUs across sites within the Inambari and Huallaga, **C**, **D** respectively.

**Figure 9.**
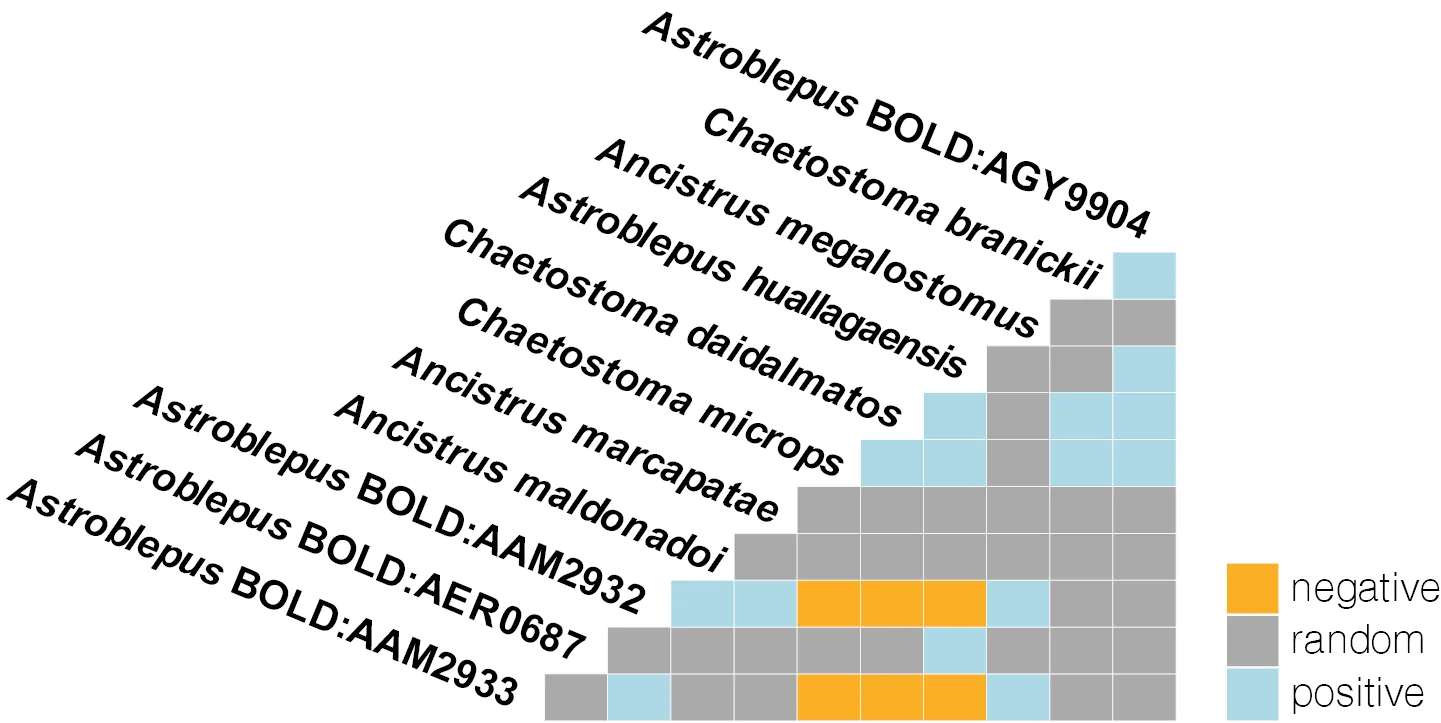
Matrix of co-occurrence among MOTUs based on the probabilistic model of Veech (2013). Only MOTUs involved in pairwise comparisons whose co-occurrence patterns significantly depart from expected at random at included.

**Figure 10.**
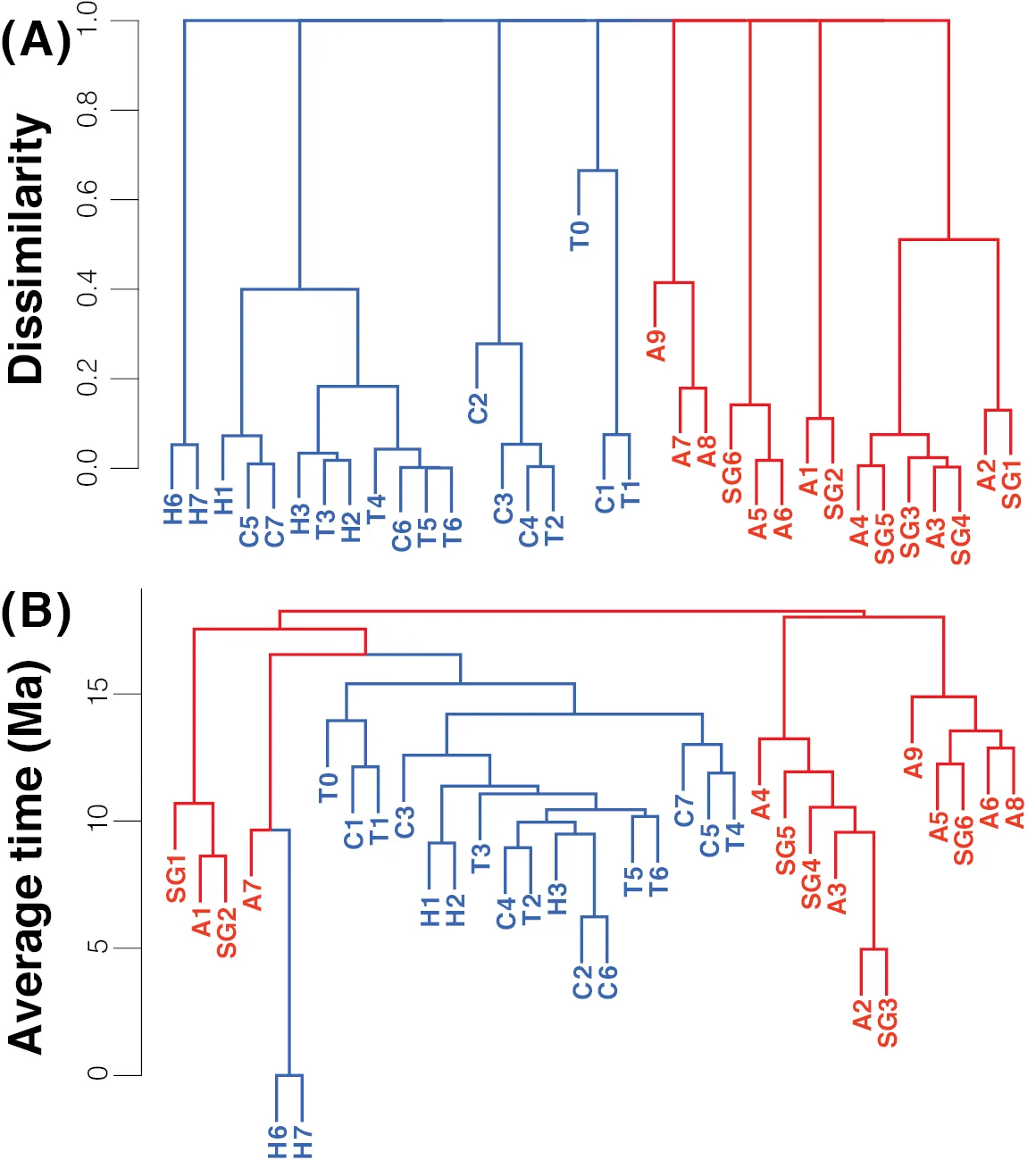
Analysis of beta-diversity among sites based on hierarchical clustering using matrices of the dissimilarity index among sites (**A**) or the average phylogenetic distance among sites (**B**).

Species richness varied among sites, ranging from three sites with a single MOTU to two sites with seven MOTUs in the Inambari, and from two sites with a single MOTU to one site with six MOTUs in the Huallaga (Fig. 8). MOTUs commonness also varies considerably, with MOTUs occurrence ranging from four MOTUs in only one site to three MOTUs found in > 10 sites in the Inambari, and from three MOTUs restricted to a single site to two MOTUs distributed in nine sites in the Huallaga (Fig. 8). The analysis of MOTUs co-occurrence using the probabilistic model of Veech (2013) revealed that only 21 of the 351 pairs of MOTUs exhibited nonrandom patterns (Fig. 9). The hierarchical clustering based on sites dissimilarity in MOTUs occurrence recovered eight clusters that shared no MOTUs (Fig. 10), and each grouping sites from the same watershed (Inambari or Huallaga). A similar spatial structure was observed in the hierarchical clustering based on average phylogenetic distances among sites, with sites within each watershed clustering together.

Indexes of species dominance (*D*, *d*) indicated that the diversity within sites was generally high, with the probability of sampling the same species twice below 0.5 in most sites. The *J* index also showed generally high values, ranging from 0 to 2.038 (Table 3). Analyses of phylogenetic relatedness of MOTUs within sites showed no significant departures from expected by chance of Mean Phylogenetic Distance (MPD) and Mean Nearest Taxon Distance (MNTD) in most sites. Exceptions included sites C4, C6, T2, T3, T5, T6 and H3 for MPD, and C6, T2, T5, and T6 for MNTD, where an excess of closely related species was detected (Table 3).

**Table 3.** Summary statistics of the community structure analysis including the number of MOTUs included in the computations (NMOTUs), the total number of individuals (N Individuals), the Simpson index of Dominance (D), the Margalef index (d), the Pielou index (J), the observed Mean Phylogenetic Distance (MPDobs), the average Mean Random Phylogenetic Distance estimated based on random assortment of species (MPDsim), the standardized deviation of MPD from expected at random (MPD z) and its significance level (p), the Mean Nearest Taxon Distance (MNTD) observed (MNTDobs), the average of expected values based on random assortment of species (MNTDsim), the standardized deviation of MNTD from expected at random (MNTD z) and its significance (p). A total of 1000 random rearrangements of MOTUs per site were conducted to estimate the significance of the departures observed.

		**N MOTUs**	**N Individuals**	***D***	***d***	***J***	**MPDobs**	**MPDsim**	**MPD z**	***p***	**MNTDobs**	**MNTDsim**	**MNTD z**	***p***
**Inambari**	**A1**	1	5	1.000	0.000	–	–	–	–	–	–	–	–	–
	**A2**	1	10	1.000	0.000	–	–	–	–	–	–	–	–	–
	**A3**	3	8	0.344	0.962	0.985	15.810	16.119	-0.159	0.352	12.867	14.869	-0.777	0.173
	**A4**	5	48	0.374	1.033	0.737	15.766	16.107	-0.296	0.310	10.523	12.970	-1.232	0.118
	**A5**	6	17	0.246	1.765	0.889	14.170	16.158	-2.116	0.042	8.479	12.326	-2.130	0.027
	**A6**	7	19	0.241	2.038	0.838	15.651	16.158	-0.628	0.221	11.625	11.719	-0.058	0.441
	**A7**	1	2	1.000	0.000	–	–	–	–	–	–	–	–	–
	**A8**	3	6	0.333	1.116	1.000	14.676	16.217	-0.836	0.150	10.599	14.789	-1.536	0.088
	**A9**	7	28	0.202	1.801	0.885	16.658	16.162	0.639	0.687	10.556	11.596	-0.635	0.266
	**SG1**	2	2	0.500	1.443	1.000	17.248	16.247	0.310	0.481	17.248	16.334	0.292	0.478
	**SG2**	2	4	0.625	0.721	0.811	17.248	16.219	0.323	0.477	17.248	16.156	0.335	0.488
	**SG3**	2	3	0.556	0.910	0.918	9.924	16.199	-1.903	0.063	9.924	16.196	-1.972	0.046
	**SG4**	3	7	0.347	1.028	0.982	15.810	16.119	-0.159	0.352	12.867	14.869	-0.777	0.173
	**SG5**	3	5	0.360	1.243	0.960	15.810	16.054	-0.118	0.348	12.867	14.745	-0.705	0.184
	**SG6**	2	4	0.500	0.721	1.000	18.753	16.078	0.839	0.834	18.753	16.081	0.778	0.818
**Huallaga**	**C1**	2	17	0.516	0.353	0.977	16.550	16.307	0.076	0.324	16.550	16.237	0.095	0.331
	**C2**	1	7	1.000	0.000	–	–	–	–	–	–	–	–	–
	**C3**	4	14	0.388	1.137	0.787	15.626	16.207	-0.441	0.260	12.906	13.634	-0.314	0.326
	**C4**	4	12	0.292	1.207	0.944	12.107	16.148	-2.990	0.014*	8.948	13.645	-2.031	0.041
	**C5**	3	5	0.360	1.243	0.960	15.864	16.191	-0.171	0.342	15.179	14.830	0.134	0.523
	**C6**	4	13	0.325	1.170	0.880	10.533	16.218	-4.184	0.005*	7.038	13.695	-2.906	0.005*
	**C7**	5	23	0.282	1.276	0.884	16.087	16.189	-0.095	0.373	11.614	12.837	-0.587	0.261
	**T0**	3	26	0.506	0.614	0.754	15.515	16.234	-0.365	0.270	14.480	14.662	-0.068	0.394
	**T1**	5	28	0.347	1.200	0.813	15.108	16.209	-1.041	0.134	12.677	12.806	-0.061	0.444
	**T2**	5	14	0.327	1.516	0.827	10.649	16.227	-5.374	0.001*	7.413	12.886	-2.653	0.005*
	**T3**	5	15	0.244	1.477	0.926	13.219	16.250	-2.819	0.021*	9.080	12.877	-1.825	0.051
	**T4**	5	22	0.231	1.294	0.949	14.248	16.167	-1.642	0.074	10.215	12.927	-1.399	0.087
	**T5**	5	15	0.262	1.477	0.907	12.734	16.208	-3.105	0.014*	8.118	12.884	-2.373	0.016*
	**T6**	5	16	0.281	1.443	0.884	12.734	16.208	-3.105	0.014*	8.118	12.884	-2.373	0.016*
	**H1**	2	9	0.803	0.455	0.503	16.550	16.161	0.119	0.338	16.550	16.152	0.121	0.344
	**H2**	3	12	0.458	0.805	0.808	12.755	16.307	-1.887	0.046	8.960	14.682	-2.100	0.052
	**H3**	6	24	0.257	1.573	0.856	10.902	16.227	-5.899	0.001*	7.112	12.334	-2.931	0.003*
	**H6**	1	3	1.000	0.000	–	–	–	–	–	–	–	–	–
	**H7**	1	2	1.000	0.000	–	–	–	–	–	–	–	–	–

## Discussion

Our study confirms the benefits of integrating DNA barcoding into the taxonomic workflow of biodiversity inventories in species-rich, yet poorly documented, ichthyofaunas, by accelerating species delimitation procedure in cases of undocumented diversity. It further provides an updated assessment of Siluriformes diversity in the Andes through the generation of new DNA barcode records, resulting in a reference library including sequences from 446 individuals from the headwaters of two major Amazonian tributaries, namely the Upper Amazon (Huallaga) and Madeira (Inambari) rivers. DNA-based species delimitation methods largely converged on the delineation of 27 MOTUs. The five methods were highly concordant, with only a few exceptions, and a barcoding gap was observed among all 27 MOTUs (Tables 1, 2). These results support the value of combining several species delimitation methods and relying on a consensus rather a single method when assessing the robustness of delimitation schemes (Kekkonen et al. 2015; Blair and Bryson 2017; Shen et al. 2019; Delrieu‐Trottin et al. 2020; Sholihah et al. 2020). The proportion of MOTUs identified only to the genus level was high for both families, with a total of 15 MOTUs not assigned to the species level (Table 2). This pattern is consistent with previous molecular studies of Siluriformes in mountainous regions of the Amazon basin (Schaefer et al. 2011; Prizon et al. 2017; de Borba et al. 2019; Reis and De Pinna 2023). However, this trend contrasts with studies of lowland lineages, where untapped diversity is typically detected across broader spatial scales among Amazonian fishes (e.g., Limeira et al. 2024). In addition, several closely related MOTUs with low genetic divergence were detected in both Loricariidae and Astroblepidae (Fig. 5, Table 1), with species exhibiting K2P genetic distances of 2% or less to their closest relatives in the dataset. This pattern was particularly evident in *Ancistrus tamboensis* and *Chaetostoma stroumpoulos*, for which nearest-neighbor distances were close to 1%, as well as in several *Astroblepus*MOTUs with distances approaching 2%. Taken together, the results of this molecular inventory suggest that Siluriformes diversity in the Andes is likely underestimated, with several MOTUs representing potential candidate species, a pattern consistent with the ongoing increase in species description published in recent years in the region (Urbano‐Bonilla and Ballen 2021; Faustino‐Fuster and de Souza 2022; Meza-Vargas et al. 2022; Faustino-Fuster et al. 2024; Neuhaus et al. 2024).

The present study highlights that most MOTUs exhibit particularly small phylogenetic distances to each other, suggesting that *in situ* diversification has been an important evolutionary driver among Siluriformes in the Andes. This pattern was expected given the ancient and complex geological history of the Andean region, which experienced an intense orogenic activity during the Miocene (Schellart 2017; Baby et al. 2025) and provided multiple opportunities for *in situ* diversification through repeated reshaping of Neotropical watersheds (Lundberg 1998; Lundberg et al. 1998; Hubert and Renno 2006; Albert and Reis 2011). The Andes represent a highly fragmented landscape due to their extreme elevation gradients, which promote geographic isolation and foster endemism (Fernandes et al. 2004; Schaefer et al. 2011; Elías et al. 2023). Our results further suggest that the increase in species diversity in the Peruvian Andes likely resulted from a combination of immigration and *in situ* diversification during the last 10 Ma (Fig. 5). Patterns of species distribution and turnover, however, point to high level of endemism across elevational gradients, with little overlap in MOTUs composition among sites at different elevation, as well as among different watersheds or tributaries. This pattern suggests an important contribution of *in situ* diversification in the assembly of Andean catfish assemblages, likely driven by wide range of ecological conditions across the Andean elevational gradient (Lujan et al. 2013) and the complex geological history of the region (Schellart 2017). The detection of clusters of sites at small spatial scales within rivers, with no MOTUs shared with neighboring sites, suggests that endemism is likely structured by sharp clines in MOTUs range distributions. Most MOTUs were restricted to only a few sites within both the Inambari and Huallaga watersheds, a pattern consistent with very low connectivity of the upstream sections of the different river systems (Figs 5, 8). This result was further supported by the detection of non-overlapping MOTUs with significant departures from random patterns of co-occurrence (Fig. 9). Together these findings indicate that Siluriformes species tend to occupy narrow elevational ranges along the Andean slope, consistent with the presence of sharp ecological transitions in Andean aquatic ecosystems (Lujan et al. 2013). The analyses of phylogenetic relatedness among MOTUs within sites further revealed an excess of closely related species, as both MPD and MNRT values were significantly smaller than expected under random assembly for multiple sites. These results indicate that catfish communities are not resulting from the random sorting of phylogenetic lineages in the regional species pool as they present an excess of closely related species, particularly in the Huallaga, a pattern consistent with the dominance of *in situ* diversification during community assembly along elevational gradients (Emerson and Gillespie 2008; Hubert et al. 2015). This mode of community assembly is further supported by the detection of non-random patterns of species aggregation, suggesting ecological specialization across elevational gradients. Overall, these patterns indicate that species diversification and community assembly in Andean Siluriformes are driven by the shared mechanisms, including limited connectivity, landscape fragmentation, and ecological specialization along the Andean slope.

## Conclusions

The present study highlights important knowledge gaps among Andean Siluriformes and confirms the utility of standardized DNA-based species delimitation methods in aiding biodiversity inventories. When applied to poorly surveyed ichthyofaunas, such as those of the Andes, these approaches facilitate the discovery of previously unrecognized biodiversity and help identify priorities for further taxonomic research. In this study, a total of 27 MOTUs were detected, of which 15 could not be identified based to species level based on an initial screening of their morphology and therefore require re-examination of their diagnostic characters and potentially a formal description. This number of potential new species is high and likely still underestimated for several groups, particularly *Astroblepus*, further emphasizing the need to improve our knowledge of Andean fish diversity. Finally, the present study reveals high levels of endemism and underscores the importance of *in situ* diversification in the assembly of catfish communities along the Andean slope.

## Additional information

### Artificial Intelligence (AI) use

Regarding the use of AI in the preparation of this manuscript, the authors declare the following:

ChatGPT was used to revise grammar and synthax in english

### Author ORCIDs

M. Hidalgo https://orcid.org/0000-0002-0071-5159

G. Estivals https://orcid.org/0000-0001-5568-3548

D. Faustino-Fuster https://orcid.org/0000-0002-1445-3495

V. Meza-Vargas https://orcid.org/0000-0002-1041-1271

R. Britzke R https://orcid.org/0000-0002-1042-2544

D. Steinke https://orcid.org/0000-0002-8992-575X

N. Hubert https://orcid.org/0000-0001-9248-3377

### Data availability

All of the data that support the findings of this study are available in the main text or Supplementary Information.

## Supplementary materials

Supplementary material 1 Neighbor joining tree of the 446 DNA barcodes produced in BOLD Data type pdf https://binary.pensoft.net/file/1748657This dataset is made available under the Open Database License (http://opendatacommons.org/licenses/odbl/1.0/). The Open Database License (ODbL) is a license agreement intended to allow users to freely share, modify, and use this Dataset while maintaining this same freedom for others, provided that the original source and author(s) are credited. Nicolas Hubert

Supplementary material 2 Colateral data of the 446 DNA barcode records Data type xlsx Explanation note Metadata accompaying the 446 DNA barcode records including sampling information and species delimitation results. https://binary.pensoft.net/file/1748658This dataset is made available under the Open Database License (http://opendatacommons.org/licenses/odbl/1.0/). The Open Database License (ODbL) is a license agreement intended to allow users to freely share, modify, and use this Dataset while maintaining this same freedom for others, provided that the original source and author(s) are credited. Aaron De la Sota, Nicolas Hubert
