# Supplementary material for: Delimiting Andean catfish species of the families Astroblepidae and Loricariidae (Ostariophysi, Siluriformes) in the Peruvian Andes using DNA barcodes
Source: Zookeys. 2026 Aug 18;1290:59–84. doi: 10.3897/zookeys.1290.182593 (PMC13507773; doi:10.3897/zookeys.1290.182593)
Supplement: Supplementary file 1 [file zookeys-1290-059_article-182593__-s001.pdf]

BOLD TaxonID Tree

Title	:	Tree Result - DS-ANDEAN (446 records selected)
Date	:	10-Jun-2026
Data Type	:	Nucleotide
Distance Model	:	Kimura 2 Parameter
Marker	:	COI-5P
Colourization	:	[blue]=Stop Codons [red]=Contamination or misidentification

Label	:	Sample ID
Label	:	Taxon
Label	:	Barcode Cluster (BIN)

Sequence Count	:	446
Species count	:	14
Genus count	:	3
Family count	:	2
Unidentified	:	105

BIN Count	:	27

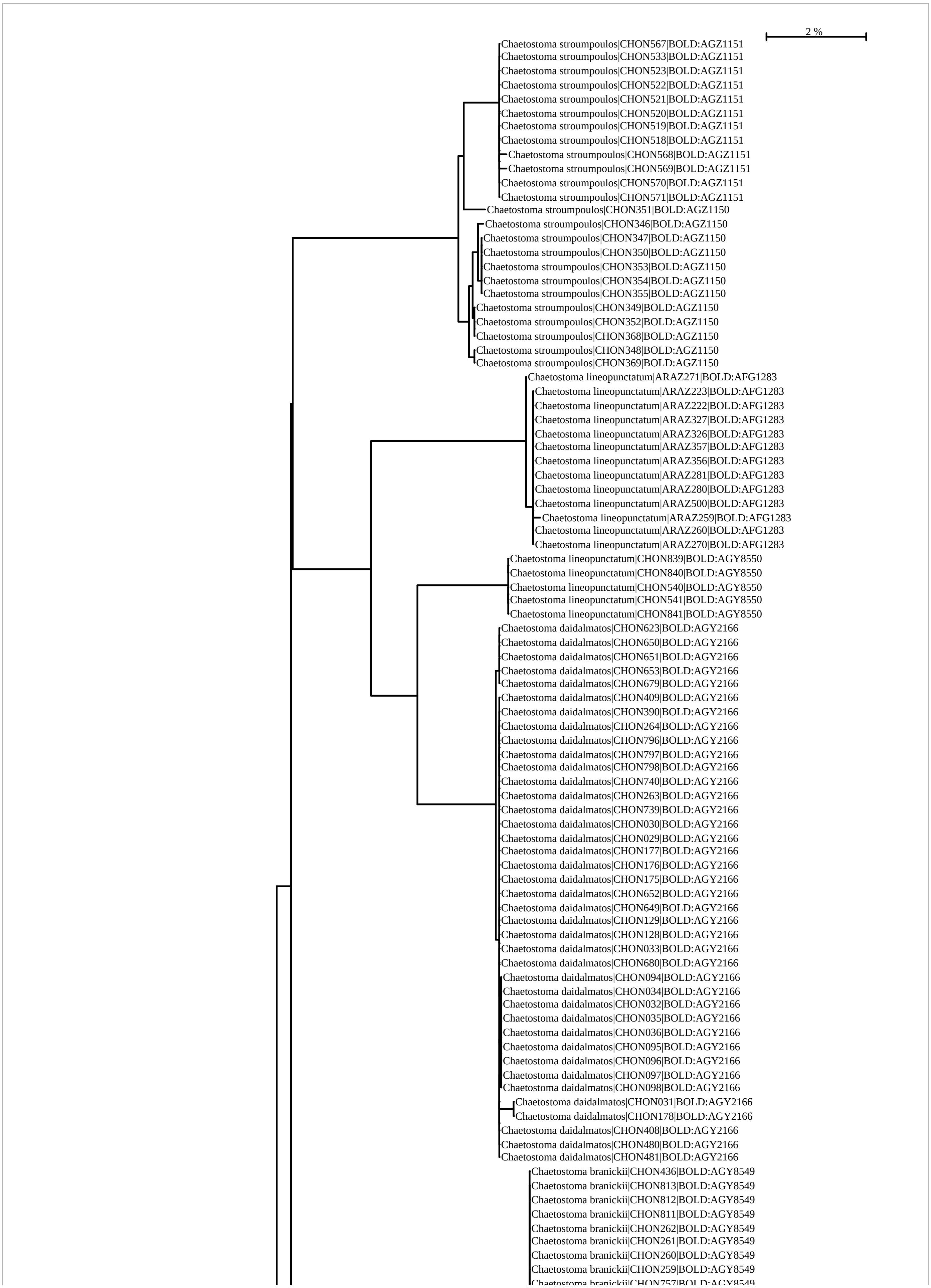
2 %
Chaetostoma stroumpoulos|CHON567|BOLD:AGZ1151
Chaetostoma stroumpoulos|CHON533|BOLD:AGZ1151
Chaetostoma stroumpoulos|CHON523|BOLD:AGZ1151
Chaetostoma stroumpoulos|CHON522|BOLD:AGZ1151
Chaetostoma stroumpoulos|CHON521|BOLD:AGZ1151
Chaetostoma stroumpoulos|CHON520|BOLD:AGZ1151
Chaetostoma stroumpoulos|CHON519|BOLD:AGZ1151
Chaetostoma stroumpoulos|CHON518|BOLD:AGZ1151
Chaetostoma stroumpoulos|CHON568|BOLD:AGZ1151
Chaetostoma stroumpoulos|CHON569|BOLD:AGZ1151
Chaetostoma stroumpoulos|CHON570|BOLD:AGZ1151
Chaetostoma stroumpoulos|CHON571|BOLD:AGZ1151
Chaetostoma stroumpoulos|CHON351|BOLD:AGZ1150
Chaetostoma stroumpoulos|CHON346|BOLD:AGZ1150
Chaetostoma stroumpoulos|CHON347|BOLD:AGZ1150
Chaetostoma stroumpoulos|CHON350|BOLD:AGZ1150
Chaetostoma stroumpoulos|CHON353|BOLD:AGZ1150
Chaetostoma stroumpoulos|CHON354|BOLD:AGZ1150
Chaetostoma stroumpoulos|CHON355|BOLD:AGZ1150
Chaetostoma stroumpoulos|CHON349|BOLD:AGZ1150
Chaetostoma stroumpoulos|CHON352|BOLD:AGZ1150
Chaetostoma stroumpoulos|CHON368|BOLD:AGZ1150
Chaetostoma stroumpoulos|CHON348|BOLD:AGZ1150
Chaetostoma stroumpoulos|CHON369|BOLD:AGZ1150
Chaetostoma lineopunctatum|ARAZ271|BOLD:AFG1283
Chaetostoma lineopunctatum|ARAZ223|BOLD:AFG1283
Chaetostoma lineopunctatum|ARAZ222|BOLD:AFG1283
Chaetostoma lineopunctatum|ARAZ327|BOLD:AFG1283
Chaetostoma lineopunctatum|ARAZ326|BOLD:AFG1283
Chaetostoma lineopunctatum|ARAZ357|BOLD:AFG1283
Chaetostoma lineopunctatum|ARAZ356|BOLD:AFG1283
Chaetostoma lineopunctatum|ARAZ281|BOLD:AFG1283
Chaetostoma lineopunctatum|ARAZ280|BOLD:AFG1283
Chaetostoma lineopunctatum|ARAZ500|BOLD:AFG1283
Chaetostoma lineopunctatum|ARAZ259|BOLD:AFG1283
Chaetostoma lineopunctatum|ARAZ260|BOLD:AFG1283
Chaetostoma lineopunctatum|ARAZ270|BOLD:AFG1283
Chaetostoma lineopunctatum|CHON839|BOLD:AGY8550
Chaetostoma lineopunctatum|CHON840|BOLD:AGY8550
Chaetostoma lineopunctatum|CHON540|BOLD:AGY8550
Chaetostoma lineopunctatum|CHON541|BOLD:AGY8550
Chaetostoma lineopunctatum|CHON841|BOLD:AGY8550
Chaetostoma daidalmatos|CHON623|BOLD:AGY2166
Chaetostoma daidalmatos|CHON650|BOLD:AGY2166
Chaetostoma daidalmatos|CHON651|BOLD:AGY2166
Chaetostoma daidalmatos|CHON653|BOLD:AGY2166
Chaetostoma daidalmatos|CHON679|BOLD:AGY2166
Chaetostoma daidalmatos|CHON409|BOLD:AGY2166
Chaetostoma daidalmatos|CHON390|BOLD:AGY2166
Chaetostoma daidalmatos|CHON264|BOLD:AGY2166
Chaetostoma daidalmatos|CHON796|BOLD:AGY2166
Chaetostoma daidalmatos|CHON797|BOLD:AGY2166
Chaetostoma daidalmatos|CHON798|BOLD:AGY2166
Chaetostoma daidalmatos|CHON740|BOLD:AGY2166
Chaetostoma daidalmatos|CHON263|BOLD:AGY2166
Chaetostoma daidalmatos|CHON739|BOLD:AGY2166
Chaetostoma daidalmatos|CHON030|BOLD:AGY2166
Chaetostoma daidalmatos|CHON029|BOLD:AGY2166
Chaetostoma daidalmatos|CHON177|BOLD:AGY2166
Chaetostoma daidalmatos|CHON176|BOLD:AGY2166
Chaetostoma daidalmatos|CHON175|BOLD:AGY2166
Chaetostoma daidalmatos|CHON652|BOLD:AGY2166
Chaetostoma daidalmatos|CHON649|BOLD:AGY2166
Chaetostoma daidalmatos|CHON129|BOLD:AGY2166
Chaetostoma daidalmatos|CHON128|BOLD:AGY2166
Chaetostoma daidalmatos|CHON033|BOLD:AGY2166
Chaetostoma daidalmatos|CHON680|BOLD:AGY2166
Chaetostoma daidalmatos|CHON094|BOLD:AGY2166
Chaetostoma daidalmatos|CHON034|BOLD:AGY2166
Chaetostoma daidalmatos|CHON032|BOLD:AGY2166
Chaetostoma daidalmatos|CHON035|BOLD:AGY2166
Chaetostoma daidalmatos|CHON036|BOLD:AGY2166
Chaetostoma daidalmatos|CHON095|BOLD:AGY2166
Chaetostoma daidalmatos|CHON096|BOLD:AGY2166
Chaetostoma daidalmatos|CHON097|BOLD:AGY2166
Chaetostoma daidalmatos|CHON098|BOLD:AGY2166
Chaetostoma daidalmatos|CHON031|BOLD:AGY2166
Chaetostoma daidalmatos|CHON178|BOLD:AGY2166
Chaetostoma daidalmatos|CHON408|BOLD:AGY2166
Chaetostoma daidalmatos|CHON480|BOLD:AGY2166
Chaetostoma daidalmatos|CHON481|BOLD:AGY2166
Chaetostoma branickii|CHON436|BOLD:AGY8549
Chaetostoma branickii|CHON813|BOLD:AGY8549
Chaetostoma branickii|CHON812|BOLD:AGY8549
Chaetostoma branickii|CHON811|BOLD:AGY8549
Chaetostoma branickii|CHON262|BOLD:AGY8549
Chaetostoma branickii|CHON261|BOLD:AGY8549
Chaetostoma branickii|CHON260|BOLD:AGY8549
Chaetostoma branickii|CHON259|BOLD:AGY8549
Chaetostoma branickii|CHON757|BOLD:AGY8549

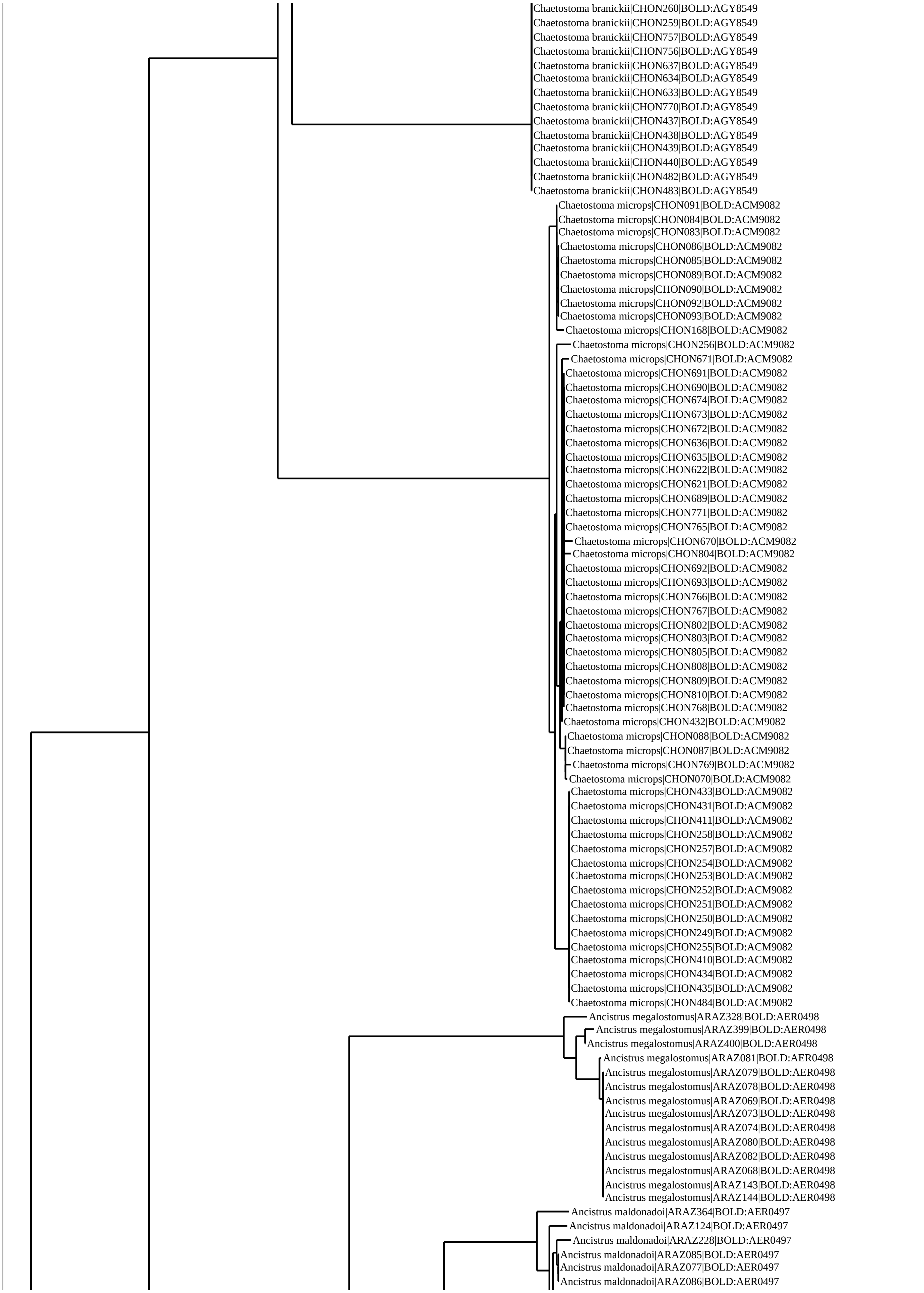
Chaetostoma branickii|CHON260|BOLD:AGY8549
Chaetostoma branickii|CHON259|BOLD:AGY8549
Chaetostoma branickii|CHON757|BOLD:AGY8549
Chaetostoma branickii|CHON756|BOLD:AGY8549
Chaetostoma branickii|CHON637|BOLD:AGY8549
Chaetostoma branickii|CHON634|BOLD:AGY8549
Chaetostoma branickii|CHON633|BOLD:AGY8549
Chaetostoma branickii|CHON770|BOLD:AGY8549
Chaetostoma branickii|CHON437|BOLD:AGY8549
Chaetostoma branickii|CHON438|BOLD:AGY8549
Chaetostoma branickii|CHON439|BOLD:AGY8549
Chaetostoma branickii|CHON440|BOLD:AGY8549
Chaetostoma branickii|CHON482|BOLD:AGY8549
Chaetostoma branickii|CHON483|BOLD:AGY8549
Chaetostoma microps|CHON091|BOLD:ACM9082
Chaetostoma microps|CHON084|BOLD:ACM9082
Chaetostoma microps|CHON083|BOLD:ACM9082
Chaetostoma microps|CHON086|BOLD:ACM9082
Chaetostoma microps|CHON085|BOLD:ACM9082
Chaetostoma microps|CHON089|BOLD:ACM9082
Chaetostoma microps|CHON090|BOLD:ACM9082
Chaetostoma microps|CHON092|BOLD:ACM9082
Chaetostoma microps|CHON093|BOLD:ACM9082
Chaetostoma microps|CHON168|BOLD:ACM9082
Chaetostoma microps|CHON256|BOLD:ACM9082
Chaetostoma microps|CHON671|BOLD:ACM9082
Chaetostoma microps|CHON691|BOLD:ACM9082
Chaetostoma microps|CHON690|BOLD:ACM9082
Chaetostoma microps|CHON674|BOLD:ACM9082
Chaetostoma microps|CHON673|BOLD:ACM9082
Chaetostoma microps|CHON672|BOLD:ACM9082
Chaetostoma microps|CHON636|BOLD:ACM9082
Chaetostoma microps|CHON635|BOLD:ACM9082
Chaetostoma microps|CHON622|BOLD:ACM9082
Chaetostoma microps|CHON621|BOLD:ACM9082
Chaetostoma microps|CHON689|BOLD:ACM9082
Chaetostoma microps|CHON771|BOLD:ACM9082
Chaetostoma microps|CHON765|BOLD:ACM9082
Chaetostoma microps|CHON670|BOLD:ACM9082
Chaetostoma microps|CHON804|BOLD:ACM9082
Chaetostoma microps|CHON692|BOLD:ACM9082
Chaetostoma microps|CHON693|BOLD:ACM9082
Chaetostoma microps|CHON766|BOLD:ACM9082
Chaetostoma microps|CHON767|BOLD:ACM9082
Chaetostoma microps|CHON802|BOLD:ACM9082
Chaetostoma microps|CHON803|BOLD:ACM9082
Chaetostoma microps|CHON805|BOLD:ACM9082
Chaetostoma microps|CHON808|BOLD:ACM9082
Chaetostoma microps|CHON809|BOLD:ACM9082
Chaetostoma microps|CHON810|BOLD:ACM9082
Chaetostoma microps|CHON768|BOLD:ACM9082
Chaetostoma microps|CHON432|BOLD:ACM9082
Chaetostoma microps|CHON088|BOLD:ACM9082
Chaetostoma microps|CHON087|BOLD:ACM9082
Chaetostoma microps|CHON769|BOLD:ACM9082
Chaetostoma microps|CHON070|BOLD:ACM9082
Chaetostoma microps|CHON433|BOLD:ACM9082
Chaetostoma microps|CHON431|BOLD:ACM9082
Chaetostoma microps|CHON411|BOLD:ACM9082
Chaetostoma microps|CHON258|BOLD:ACM9082
Chaetostoma microps|CHON257|BOLD:ACM9082
Chaetostoma microps|CHON254|BOLD:ACM9082
Chaetostoma microps|CHON253|BOLD:ACM9082
Chaetostoma microps|CHON252|BOLD:ACM9082
Chaetostoma microps|CHON251|BOLD:ACM9082
Chaetostoma microps|CHON250|BOLD:ACM9082
Chaetostoma microps|CHON249|BOLD:ACM9082
Chaetostoma microps|CHON255|BOLD:ACM9082
Chaetostoma microps|CHON410|BOLD:ACM9082
Chaetostoma microps|CHON434|BOLD:ACM9082
Chaetostoma microps|CHON435|BOLD:ACM9082
Chaetostoma microps|CHON484|BOLD:ACM9082
Ancistrus megalostomus|ARAZ328|BOLD:AER0498
Ancistrus megalostomus|ARAZ399|BOLD:AER0498
Ancistrus megalostomus|ARAZ400|BOLD:AER0498
Ancistrus megalostomus|ARAZ081|BOLD:AER0498
Ancistrus megalostomus|ARAZ079|BOLD:AER0498
Ancistrus megalostomus|ARAZ078|BOLD:AER0498
Ancistrus megalostomus|ARAZ069|BOLD:AER0498
Ancistrus megalostomus|ARAZ073|BOLD:AER0498
Ancistrus megalostomus|ARAZ074|BOLD:AER0498
Ancistrus megalostomus|ARAZ080|BOLD:AER0498
Ancistrus megalostomus|ARAZ082|BOLD:AER0498
Ancistrus megalostomus|ARAZ068|BOLD:AER0498
Ancistrus megalostomus|ARAZ143|BOLD:AER0498
Ancistrus megalostomus|ARAZ144|BOLD:AER0498
Ancistrus maldonadoi|ARAZ364|BOLD:AER0497
Ancistrus maldonadoi|ARAZ124|BOLD:AER0497
Ancistrus maldonadoi|ARAZ228|BOLD:AER0497
Ancistrus maldonadoi|ARAZ085|BOLD:AER0497
Ancistrus maldonadoi|ARAZ077|BOLD:AER0497
Ancistrus maldonadoi|ARAZ086|BOLD:AER0497

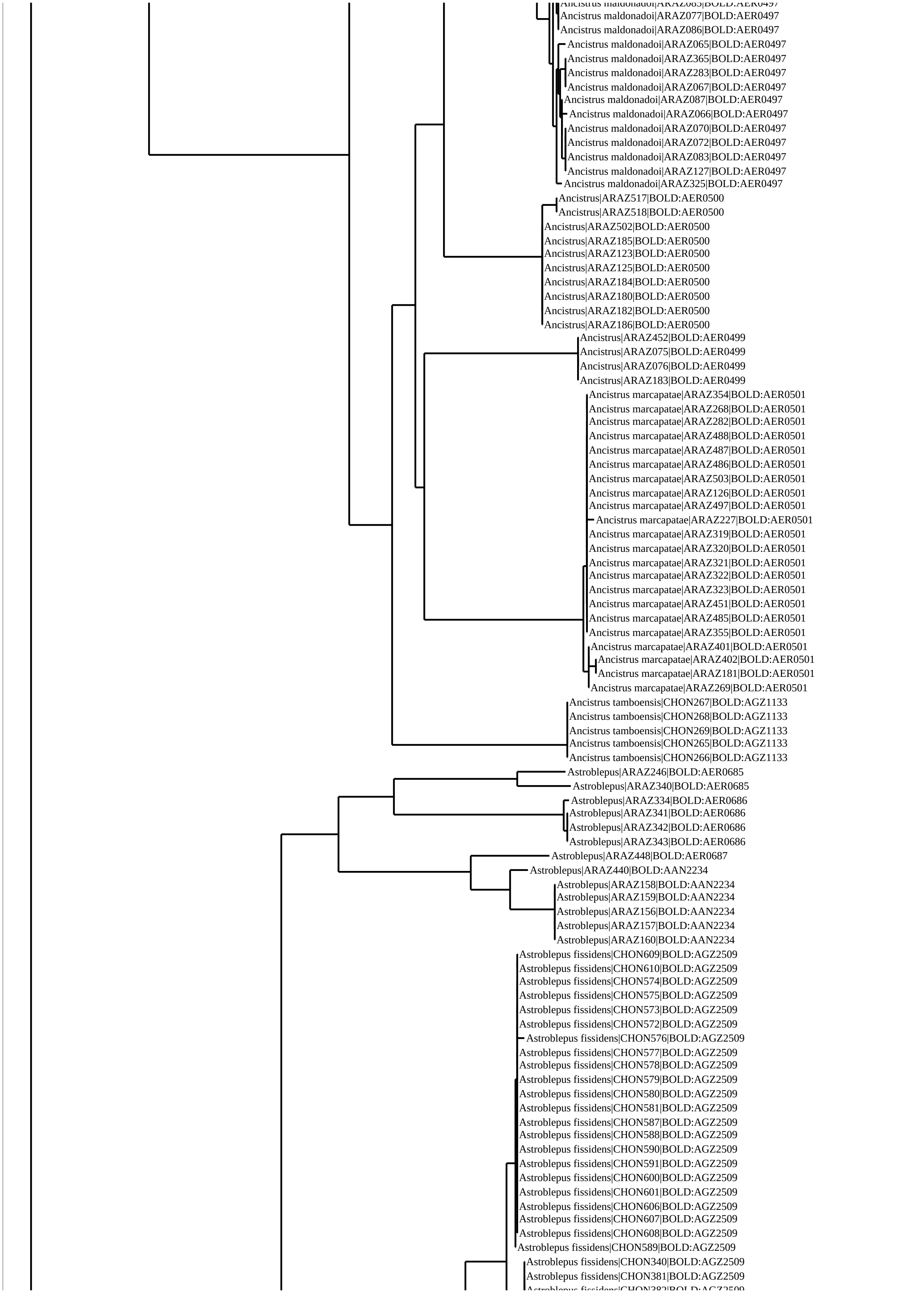
Ancistrus maldonadoi|ARAZ077|BOLD:AER0497
Ancistrus maldonadoi|ARAZ086|BOLD:AER0497
Ancistrus maldonadoi|ARAZ065|BOLD:AER0497
Ancistrus maldonadoi|ARAZ365|BOLD:AER0497
Ancistrus maldonadoi|ARAZ283|BOLD:AER0497
Ancistrus maldonadoi|ARAZ067|BOLD:AER0497
Ancistrus maldonadoi|ARAZ087|BOLD:AER0497
Ancistrus maldonadoi|ARAZ066|BOLD:AER0497
Ancistrus maldonadoi|ARAZ070|BOLD:AER0497
Ancistrus maldonadoi|ARAZ072|BOLD:AER0497
Ancistrus maldonadoi|ARAZ083|BOLD:AER0497
Ancistrus maldonadoi|ARAZ127|BOLD:AER0497
Ancistrus maldonadoi|ARAZ325|BOLD:AER0497
Ancistrus|ARAZ517|BOLD:AER0500
Ancistrus|ARAZ518|BOLD:AER0500
Ancistrus|ARAZ502|BOLD:AER0500
Ancistrus|ARAZ185|BOLD:AER0500
Ancistrus|ARAZ123|BOLD:AER0500
Ancistrus|ARAZ125|BOLD:AER0500
Ancistrus|ARAZ184|BOLD:AER0500
Ancistrus|ARAZ180|BOLD:AER0500
Ancistrus|ARAZ182|BOLD:AER0500
Ancistrus|ARAZ186|BOLD:AER0500
Ancistrus|ARAZ452|BOLD:AER0499
Ancistrus|ARAZ075|BOLD:AER0499
Ancistrus|ARAZ076|BOLD:AER0499
Ancistrus|ARAZ183|BOLD:AER0499
Ancistrus marcapatae|ARAZ354|BOLD:AER0501
Ancistrus marcapatae|ARAZ268|BOLD:AER0501
Ancistrus marcapatae|ARAZ282|BOLD:AER0501
Ancistrus marcapatae|ARAZ488|BOLD:AER0501
Ancistrus marcapatae|ARAZ487|BOLD:AER0501
Ancistrus marcapatae|ARAZ486|BOLD:AER0501
Ancistrus marcapatae|ARAZ503|BOLD:AER0501
Ancistrus marcapatae|ARAZ126|BOLD:AER0501
Ancistrus marcapatae|ARAZ497|BOLD:AER0501
Ancistrus marcapatae|ARAZ227|BOLD:AER0501
Ancistrus marcapatae|ARAZ319|BOLD:AER0501
Ancistrus marcapatae|ARAZ320|BOLD:AER0501
Ancistrus marcapatae|ARAZ321|BOLD:AER0501
Ancistrus marcapatae|ARAZ322|BOLD:AER0501
Ancistrus marcapatae|ARAZ323|BOLD:AER0501
Ancistrus marcapatae|ARAZ451|BOLD:AER0501
Ancistrus marcapatae|ARAZ485|BOLD:AER0501
Ancistrus marcapatae|ARAZ355|BOLD:AER0501
Ancistrus marcapatae|ARAZ401|BOLD:AER0501
Ancistrus marcapatae|ARAZ402|BOLD:AER0501
Ancistrus marcapatae|ARAZ181|BOLD:AER0501
Ancistrus marcapatae|ARAZ269|BOLD:AER0501
Ancistrus tamboensis|CHON267|BOLD:AGZ1133
Ancistrus tamboensis|CHON268|BOLD:AGZ1133
Ancistrus tamboensis|CHON269|BOLD:AGZ1133
Ancistrus tamboensis|CHON265|BOLD:AGZ1133
Ancistrus tamboensis|CHON266|BOLD:AGZ1133
Astroblepus|ARAZ246|BOLD:AER0685
Astroblepus|ARAZ340|BOLD:AER0685
Astroblepus|ARAZ334|BOLD:AER0686
Astroblepus|ARAZ341|BOLD:AER0686
Astroblepus|ARAZ342|BOLD:AER0686
Astroblepus|ARAZ343|BOLD:AER0686
Astroblepus|ARAZ448|BOLD:AER0687
Astroblepus|ARAZ440|BOLD:AAN2234
Astroblepus|ARAZ158|BOLD:AAN2234
Astroblepus|ARAZ159|BOLD:AAN2234
Astroblepus|ARAZ156|BOLD:AAN2234
Astroblepus|ARAZ157|BOLD:AAN2234
Astroblepus|ARAZ160|BOLD:AAN2234
Astroblepus fissidens|CHON609|BOLD:AGZ2509
Astroblepus fissidens|CHON610|BOLD:AGZ2509
Astroblepus fissidens|CHON574|BOLD:AGZ2509
Astroblepus fissidens|CHON575|BOLD:AGZ2509
Astroblepus fissidens|CHON573|BOLD:AGZ2509
Astroblepus fissidens|CHON572|BOLD:AGZ2509
Astroblepus fissidens|CHON576|BOLD:AGZ2509
Astroblepus fissidens|CHON577|BOLD:AGZ2509
Astroblepus fissidens|CHON578|BOLD:AGZ2509
Astroblepus fissidens|CHON579|BOLD:AGZ2509
Astroblepus fissidens|CHON580|BOLD:AGZ2509
Astroblepus fissidens|CHON581|BOLD:AGZ2509
Astroblepus fissidens|CHON587|BOLD:AGZ2509
Astroblepus fissidens|CHON588|BOLD:AGZ2509
Astroblepus fissidens|CHON590|BOLD:AGZ2509
Astroblepus fissidens|CHON591|BOLD:AGZ2509
Astroblepus fissidens|CHON600|BOLD:AGZ2509
Astroblepus fissidens|CHON601|BOLD:AGZ2509
Astroblepus fissidens|CHON606|BOLD:AGZ2509
Astroblepus fissidens|CHON607|BOLD:AGZ2509
Astroblepus fissidens|CHON608|BOLD:AGZ2509
Astroblepus fissidens|CHON589|BOLD:AGZ2509
Astroblepus fissidens|CHON340|BOLD:AGZ2509
Astroblepus fissidens|CHON381|BOLD:AGZ2509

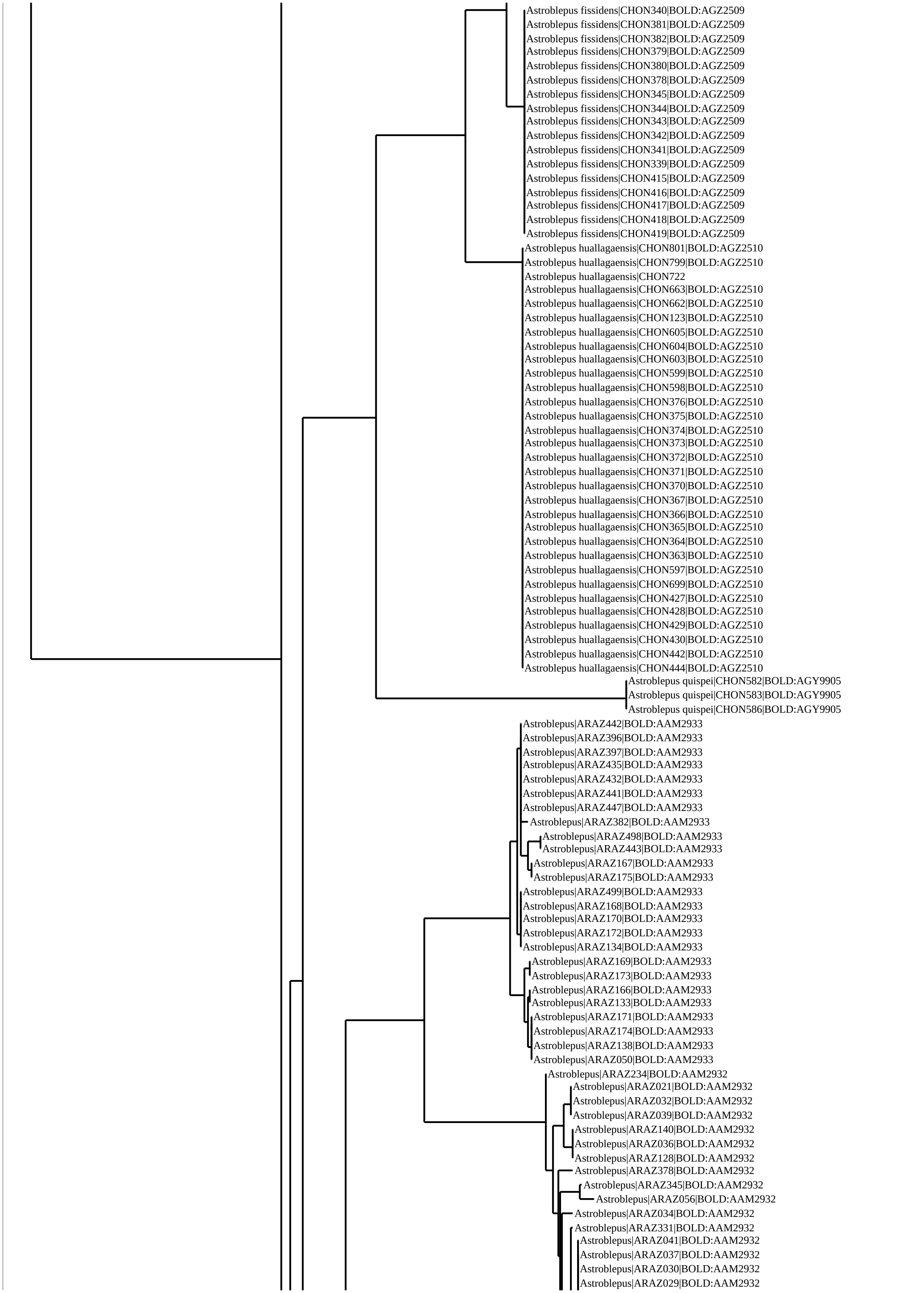
Astroblepus fissidens|CHON340|BOLD:AGZ2509
Astroblepus fissidens|CHON381|BOLD:AGZ2509
Astroblepus fissidens|CHON382|BOLD:AGZ2509
Astroblepus fissidens|CHON379|BOLD:AGZ2509
Astroblepus fissidens|CHON380|BOLD:AGZ2509
Astroblepus fissidens|CHON378|BOLD:AGZ2509
Astroblepus fissidens|CHON345|BOLD:AGZ2509
Astroblepus fissidens|CHON344|BOLD:AGZ2509
Astroblepus fissidens|CHON343|BOLD:AGZ2509
Astroblepus fissidens|CHON342|BOLD:AGZ2509
Astroblepus fissidens|CHON341|BOLD:AGZ2509
Astroblepus fissidens|CHON339|BOLD:AGZ2509
Astroblepus fissidens|CHON415|BOLD:AGZ2509
Astroblepus fissidens|CHON416|BOLD:AGZ2509
Astroblepus fissidens|CHON417|BOLD:AGZ2509
Astroblepus fissidens|CHON418|BOLD:AGZ2509
Astroblepus fissidens|CHON419|BOLD:AGZ2509
Astroblepus huallagaensis|CHON801|BOLD:AGZ2510
Astroblepus huallagaensis|CHON799|BOLD:AGZ2510
Astroblepus huallagaensis|CHON722
Astroblepus huallagaensis|CHON663|BOLD:AGZ2510
Astroblepus huallagaensis|CHON662|BOLD:AGZ2510
Astroblepus huallagaensis|CHON123|BOLD:AGZ2510
Astroblepus huallagaensis|CHON605|BOLD:AGZ2510
Astroblepus huallagaensis|CHON604|BOLD:AGZ2510
Astroblepus huallagaensis|CHON603|BOLD:AGZ2510
Astroblepus huallagaensis|CHON599|BOLD:AGZ2510
Astroblepus huallagaensis|CHON598|BOLD:AGZ2510
Astroblepus huallagaensis|CHON376|BOLD:AGZ2510
Astroblepus huallagaensis|CHON375|BOLD:AGZ2510
Astroblepus huallagaensis|CHON374|BOLD:AGZ2510
Astroblepus huallagaensis|CHON373|BOLD:AGZ2510
Astroblepus huallagaensis|CHON372|BOLD:AGZ2510
Astroblepus huallagaensis|CHON371|BOLD:AGZ2510
Astroblepus huallagaensis|CHON370|BOLD:AGZ2510
Astroblepus huallagaensis|CHON367|BOLD:AGZ2510
Astroblepus huallagaensis|CHON366|BOLD:AGZ2510
Astroblepus huallagaensis|CHON365|BOLD:AGZ2510
Astroblepus huallagaensis|CHON364|BOLD:AGZ2510
Astroblepus huallagaensis|CHON363|BOLD:AGZ2510
Astroblepus huallagaensis|CHON597|BOLD:AGZ2510
Astroblepus huallagaensis|CHON699|BOLD:AGZ2510
Astroblepus huallagaensis|CHON427|BOLD:AGZ2510
Astroblepus huallagaensis|CHON428|BOLD:AGZ2510
Astroblepus huallagaensis|CHON429|BOLD:AGZ2510
Astroblepus huallagaensis|CHON430|BOLD:AGZ2510
Astroblepus huallagaensis|CHON442|BOLD:AGZ2510
Astroblepus huallagaensis|CHON444|BOLD:AGZ2510
Astroblepus quispei|CHON582|BOLD:AGY9905
Astroblepus quispei|CHON583|BOLD:AGY9905
Astroblepus quispei|CHON586|BOLD:AGY9905
Astroblepus|ARAZ442|BOLD:AAM2933
Astroblepus|ARAZ396|BOLD:AAM2933
Astroblepus|ARAZ397|BOLD:AAM2933
Astroblepus|ARAZ435|BOLD:AAM2933
Astroblepus|ARAZ432|BOLD:AAM2933
Astroblepus|ARAZ441|BOLD:AAM2933
Astroblepus|ARAZ447|BOLD:AAM2933
Astroblepus|ARAZ382|BOLD:AAM2933
Astroblepus|ARAZ498|BOLD:AAM2933
Astroblepus|ARAZ443|BOLD:AAM2933
Astroblepus|ARAZ167|BOLD:AAM2933
Astroblepus|ARAZ175|BOLD:AAM2933
Astroblepus|ARAZ499|BOLD:AAM2933
Astroblepus|ARAZ168|BOLD:AAM2933
Astroblepus|ARAZ170|BOLD:AAM2933
Astroblepus|ARAZ172|BOLD:AAM2933
Astroblepus|ARAZ134|BOLD:AAM2933
Astroblepus|ARAZ169|BOLD:AAM2933
Astroblepus|ARAZ173|BOLD:AAM2933
Astroblepus|ARAZ166|BOLD:AAM2933
Astroblepus|ARAZ133|BOLD:AAM2933
Astroblepus|ARAZ171|BOLD:AAM2933
Astroblepus|ARAZ174|BOLD:AAM2933
Astroblepus|ARAZ138|BOLD:AAM2933
Astroblepus|ARAZ050|BOLD:AAM2933
Astroblepus|ARAZ234|BOLD:AAM2932
Astroblepus|ARAZ021|BOLD:AAM2932
Astroblepus|ARAZ032|BOLD:AAM2932
Astroblepus|ARAZ039|BOLD:AAM2932
Astroblepus|ARAZ140|BOLD:AAM2932
Astroblepus|ARAZ036|BOLD:AAM2932
Astroblepus|ARAZ128|BOLD:AAM2932
Astroblepus|ARAZ378|BOLD:AAM2932
Astroblepus|ARAZ345|BOLD:AAM2932
Astroblepus|ARAZ056|BOLD:AAM2932
Astroblepus|ARAZ034|BOLD:AAM2932
Astroblepus|ARAZ331|BOLD:AAM2932
Astroblepus|ARAZ041|BOLD:AAM2932
Astroblepus|ARAZ037|BOLD:AAM2932
Astroblepus|ARAZ030|BOLD:AAM2932
Astroblepus|ARAZ029|BOLD:AAM2932

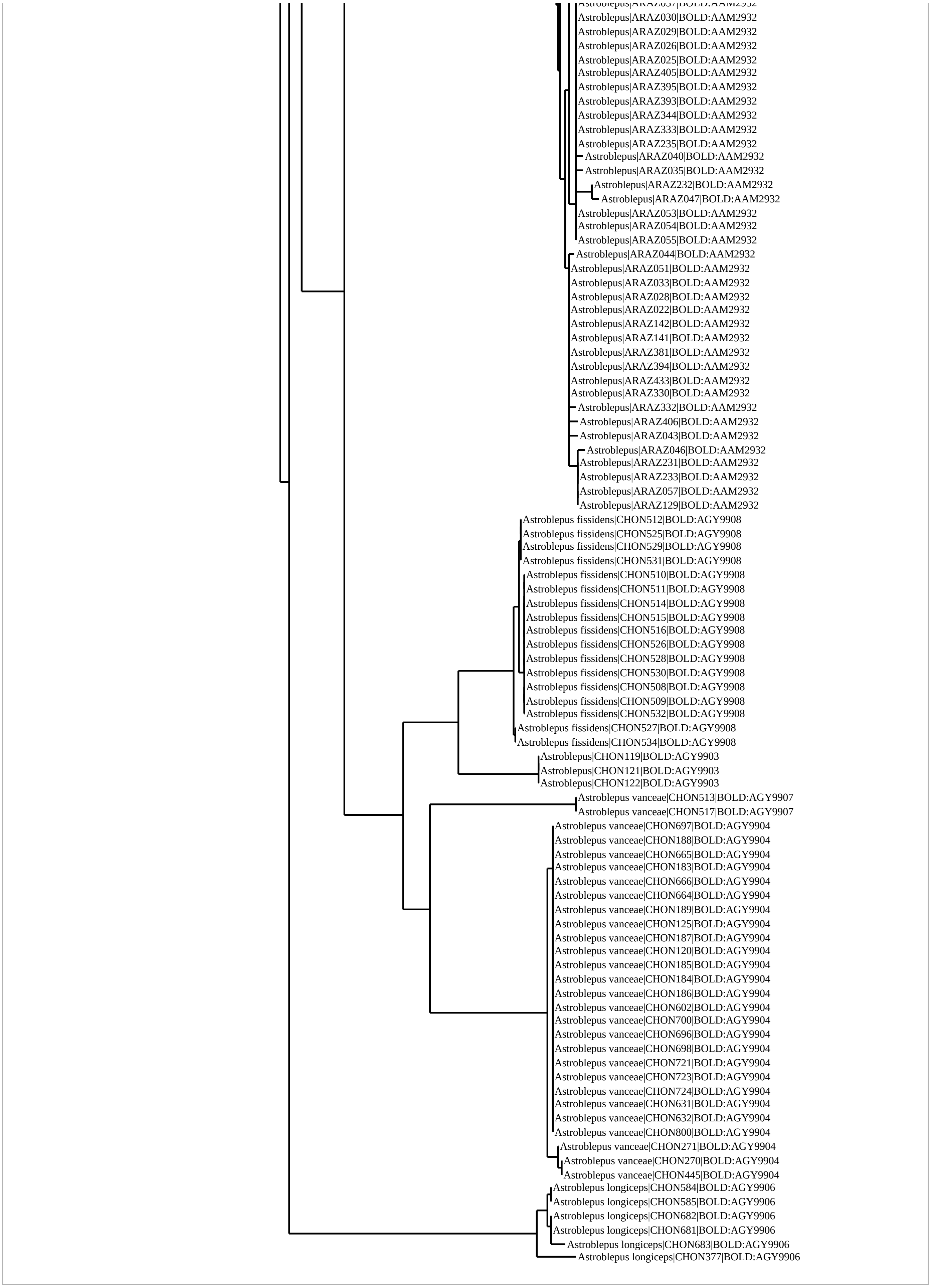
Astroblepus|ARAZ037|BOLD:AAM2932
Astroblepus|ARAZ030|BOLD:AAM2932
Astroblepus|ARAZ029|BOLD:AAM2932
Astroblepus|ARAZ026|BOLD:AAM2932
Astroblepus|ARAZ025|BOLD:AAM2932
Astroblepus|ARAZ405|BOLD:AAM2932
Astroblepus|ARAZ395|BOLD:AAM2932
Astroblepus|ARAZ393|BOLD:AAM2932
Astroblepus|ARAZ344|BOLD:AAM2932
Astroblepus|ARAZ333|BOLD:AAM2932
Astroblepus|ARAZ235|BOLD:AAM2932
Astroblepus|ARAZ040|BOLD:AAM2932
Astroblepus|ARAZ035|BOLD:AAM2932
Astroblepus|ARAZ232|BOLD:AAM2932
Astroblepus|ARAZ047|BOLD:AAM2932
Astroblepus|ARAZ053|BOLD:AAM2932
Astroblepus|ARAZ054|BOLD:AAM2932
Astroblepus|ARAZ055|BOLD:AAM2932
Astroblepus|ARAZ044|BOLD:AAM2932
Astroblepus|ARAZ051|BOLD:AAM2932
Astroblepus|ARAZ033|BOLD:AAM2932
Astroblepus|ARAZ028|BOLD:AAM2932
Astroblepus|ARAZ022|BOLD:AAM2932
Astroblepus|ARAZ142|BOLD:AAM2932
Astroblepus|ARAZ141|BOLD:AAM2932
Astroblepus|ARAZ381|BOLD:AAM2932
Astroblepus|ARAZ394|BOLD:AAM2932
Astroblepus|ARAZ433|BOLD:AAM2932
Astroblepus|ARAZ330|BOLD:AAM2932
Astroblepus|ARAZ332|BOLD:AAM2932
Astroblepus|ARAZ406|BOLD:AAM2932
Astroblepus|ARAZ043|BOLD:AAM2932
Astroblepus|ARAZ046|BOLD:AAM2932
Astroblepus|ARAZ231|BOLD:AAM2932
Astroblepus|ARAZ233|BOLD:AAM2932
Astroblepus|ARAZ057|BOLD:AAM2932
Astroblepus|ARAZ129|BOLD:AAM2932
Astroblepus fissidens|CHON512|BOLD:AGY9908
Astroblepus fissidens|CHON525|BOLD:AGY9908
Astroblepus fissidens|CHON529|BOLD:AGY9908
Astroblepus fissidens|CHON531|BOLD:AGY9908
Astroblepus fissidens|CHON510|BOLD:AGY9908
Astroblepus fissidens|CHON511|BOLD:AGY9908
Astroblepus fissidens|CHON514|BOLD:AGY9908
Astroblepus fissidens|CHON515|BOLD:AGY9908
Astroblepus fissidens|CHON516|BOLD:AGY9908
Astroblepus fissidens|CHON526|BOLD:AGY9908
Astroblepus fissidens|CHON528|BOLD:AGY9908
Astroblepus fissidens|CHON530|BOLD:AGY9908
Astroblepus fissidens|CHON508|BOLD:AGY9908
Astroblepus fissidens|CHON509|BOLD:AGY9908
Astroblepus fissidens|CHON532|BOLD:AGY9908
Astroblepus fissidens|CHON527|BOLD:AGY9908
Astroblepus fissidens|CHON534|BOLD:AGY9908
Astroblepus|CHON119|BOLD:AGY9903
Astroblepus|CHON121|BOLD:AGY9903
Astroblepus|CHON122|BOLD:AGY9903
Astroblepus vanceae|CHON513|BOLD:AGY9907
Astroblepus vanceae|CHON517|BOLD:AGY9907
Astroblepus vanceae|CHON697|BOLD:AGY9904
Astroblepus vanceae|CHON188|BOLD:AGY9904
Astroblepus vanceae|CHON665|BOLD:AGY9904
Astroblepus vanceae|CHON183|BOLD:AGY9904
Astroblepus vanceae|CHON666|BOLD:AGY9904
Astroblepus vanceae|CHON664|BOLD:AGY9904
Astroblepus vanceae|CHON189|BOLD:AGY9904
Astroblepus vanceae|CHON125|BOLD:AGY9904
Astroblepus vanceae|CHON187|BOLD:AGY9904
Astroblepus vanceae|CHON120|BOLD:AGY9904
Astroblepus vanceae|CHON185|BOLD:AGY9904
Astroblepus vanceae|CHON184|BOLD:AGY9904
Astroblepus vanceae|CHON186|BOLD:AGY9904
Astroblepus vanceae|CHON602|BOLD:AGY9904
Astroblepus vanceae|CHON700|BOLD:AGY9904
Astroblepus vanceae|CHON696|BOLD:AGY9904
Astroblepus vanceae|CHON698|BOLD:AGY9904
Astroblepus vanceae|CHON721|BOLD:AGY9904
Astroblepus vanceae|CHON723|BOLD:AGY9904
Astroblepus vanceae|CHON724|BOLD:AGY9904
Astroblepus vanceae|CHON631|BOLD:AGY9904
Astroblepus vanceae|CHON632|BOLD:AGY9904
Astroblepus vanceae|CHON800|BOLD:AGY9904
Astroblepus vanceae|CHON271|BOLD:AGY9904
Astroblepus vanceae|CHON270|BOLD:AGY9904
Astroblepus vanceae|CHON445|BOLD:AGY9904
Astroblepus longiceps|CHON584|BOLD:AGY9906
Astroblepus longiceps|CHON585|BOLD:AGY9906
Astroblepus longiceps|CHON682|BOLD:AGY9906
Astroblepus longiceps|CHON681|BOLD:AGY9906
Astroblepus longiceps|CHON683|BOLD:AGY9906
Astroblepus longiceps|CHON377|BOLD:AGY9906
